# The User Experience of Ambulatory Assessment and Mood Monitoring in Bipolar Disorder: Systematic Review and Meta-Synthesis of Qualitative Studies

**DOI:** 10.2196/71525

**Published:** 2025-10-17

**Authors:** Laurence Astill Wright, Madiha Majid, Matthew Moore, Goldie Momoh, Renee Patil, Georgina Shajan, Daljit Purewal, Shireen Patel, Richard Morriss

**Affiliations:** 1 Institute of Mental Health, University of Nottingham, Jubilee Campus, University of Nottingham Innovation Park, Triumph Road, Nottingham, NG7 2TU, United Kingdom, 44 0115 823 1294; 2 Centre for Academic Mental Health, Population Health Sciences, University of Bristol, Bristol, United Kingdom; 3 Coventry and Warwickshire Partnership NHS Trust, Coventry, United Kingdom; 4 NIHR MindTech Medical Technology Collaborative, University of Nottingham, Nottingham, United Kingdom; 5 NIHR ARC East Midlands, University of Nottingham, Nottingham, United Kingdom; 6 Nottingham NIHR Biomedical Research Centre, University of Nottingham, Nottingham, United Kingdom

**Keywords:** bipolar, bipolar disorder, depression, ecological momentary assessment, EMA, mood-tracking, mood-monitoring, self-monitoring, ambulatory assessment

## Abstract

**Background:**

Mood monitoring and ambulatory assessment hold promise for supporting self-management and data collection in bipolar disorder (BD), but the effectiveness of these depends crucially on the preferences and perspectives of those who use them. To date, these user experiences have not been systematically synthesized.

**Objective:**

This study aimed to explore and synthesize qualitative evidence on the user experience of mood monitoring and ambulatory assessment in BD, with a focus on identifying barriers and facilitators for both individuals with BD and clinicians, as well as the intended purposes of these tools.

**Methods:**

We conducted a systematic review and meta-synthesis of qualitative and mixed-methods studies reporting on ambulatory assessment and mood monitoring in BD (PROSPERO CRD42023396473). A total of 8 electronic databases were searched. Studies were appraised using established criteria for qualitative research. First- and second-order constructs were extracted, and a third-order synthesis was developed using the Noblit and Hare meta-ethnographic approach.

**Results:**

A total of 20 studies comprising 2365 participants met inclusion criteria. We identified 9 overarching themes: adverse effects, barriers to use, facilitators to use, perceived purpose, sharing with others (positive and negative), clinician concerns, clinician suggestions, and desired features. Users reported both benefits and harms. Some experienced increased insight, behavioral change, and relapse prevention skills, while others reported emotional burden, repetitive content, and worsened mood or anxiety. Preferences varied widely, but a strong, consistent emphasis was placed on personalization, including the ability to control what is tracked, when and how it is shared, and the level of engagement with health care providers. Passive data collection was often seen as less intrusive and more sustainable. Sharing data was sometimes seen as empowering, especially when it enabled communication with trusted individuals or clinicians, but also raised concerns around autonomy, misinterpretation, and privacy. Clinicians echoed many user views but raised additional concerns about liability and interpretability of data. Participants also highlighted the need for onboarding or support to aid comprehension and effective use.

**Conclusions:**

This review highlights the complexity and diversity of user experiences with ambulatory assessment and mood monitoring in BD. While many found these tools valuable for fostering insight, self-management, and relapse prevention, others found them burdensome or confronting. User engagement appears closely tied to perceived control, relevance, and personal fit. These findings underscore the need for flexible, user-centered design in future interventions. Customizability should be prioritized—including what is monitored, how feedback is delivered, and whether data is shared externally. Incorporating onboarding and adaptive feedback could help users better understand and apply their data to better self-manage. By aligning interventions more closely with user preferences and lived experience, ambulatory assessment and mood monitoring protocols may achieve greater uptake, engagement, and ultimately, a more effective intervention.

## Introduction

Self-monitoring interventions offer people with bipolar disorder (BD) an important method of self-managing their symptoms, but there are some key implementation and acceptability issues [1]. Self-monitoring is widely used by people with BD as a form of self-management [2,3], but it can also be a key element of psychological therapies for people with BD and can aid individuals to understand and manage their BD [4]. Some report improvements in detecting early warning signs of relapse, which then guide appropriate intervention, while others report insight-related behavior change [5]. Previous interventions have focused on mood and sleep, which have been demonstrated to increase time to relapse, decrease rates of hospitalization, and improve functioning [6]. Advances in digital technology have led to the emergence of new electronic mood monitoring tools designed specifically for people with BD [7]. Many of these tools use active monitoring and require users to actively input data via self-report questionnaires and surveys; others rely on automatically collected or passive data via inbuilt sensors in smartphones or via wearable devices. Some collect and use a combination of active and passive data [7]. These tools have the potential to change the way that people with BD self-manage their symptoms and quality of life more broadly. These interventions may be useful in improving prognosis, ameliorating cognitive and functional decline, and through early intervention, preventing progression. Repeated episodes may alter the course of BD, so secondary prevention of further episodes is promising in preventing the development of chronic unremitting illness—known as stage 4 in the clinical staging model representing treatment resistance and established disease [8].

There are multiple strong rationales for maximizing the user experience of these tools. These include the optimization and digitization of existing psychosocial treatments that emphasize illness management strategies and enhance coping skills, which are some of the most promising for symptom control in BD [9]. These may require further adaptation to the needs of people with BD as technology improves to enable more scalable psychological therapies [10]. Furthermore, mood monitoring interventions may improve scalability and access—as adjuncts to individual level psychological therapy for BD while also increasing the availability of empirically tested psychotherapeutic strategies for BD [6]. Currently, access to high-intensity psychological therapies is limited in some health care systems, and digital interventions promise part of the solution to providing easy-to-access and empirically supported therapies for people with BD [9-11]. The collection of active and passive data remotely and electronically may also have a significant role in the data collection to enhance and improve scientific research. This method of frequent assessment is called ambulatory assessment. Ambulatory assessment has the potential to decrease participant burden and allow more detailed frequent mood assessment to enable higher quality data of greater granularity. Although studies on the feasibility, acceptability, and validity of routine quality of life in digital self-assessment have been conducted in medical specialties [1], like oncology [12-14], this experience remains poorly understood in digital mental health [1].

The incorporation of feedback from people with BD about the user experience would likely benefit both of these slightly different, but potentially compatible use cases (ambulatory assessment for research and mood monitoring as a self-management intervention). User involvement in the user experience improves engagement with digital interventions [15]. The perspectives and preferences of individuals with BD will likely be crucial for the success of mood monitoring interventions, or for ambulatory assessment as a method of data collection [10]. For any mood monitoring intervention to be successful, it must meet the needs of people with BD and maximize participation in research-based ambulatory assessment so that the data collection must be acceptable and usable. For example, there might be potential benefits and disadvantages to particular intervention components that could be modified [16].

In this paper, we refer to the use of a mood monitoring or ambulatory assessment protocol as the particular use of mood monitoring or ambulatory assessment described in the paper and the actions that follow it. This can refer to the research protocol, the intervention, and the procedures that follow as consequences of using mood monitoring or ambulatory assessment on a particular device. The protocol does not refer to the quality of the methodology or reporting of the study. We define ambulatory assessment as a broad group of methodologies that use a high degree of frequency of assessment of mood, for example, greater than once daily and often in the individual’s natural environment in real time or near real time. Ambulatory assessment includes remote measuring technology (eg, data collected in the background by wearables, or passively), mood monitoring, and ecological momentary assessment (EMA—frequent and more intensive collection of data at random times, eg, many times per day in the participant’s environment. Our paper set out to look at acceptability issues in studies where ambulatory assessment was used as a research measure and also in those using mood monitoring as a clinical intervention, and many of these acceptability issues will be common across studies.

Many of the studies examining the user perspective of mood monitoring protocols have been limited by small sample sizes and have just focused on a particular ambulatory assessment protocol [17]. There is a paucity of research about how people with BD actually experience ambulatory assessment [17,18]. Furthermore, people with BD appear to use and incorporate these protocols into their life in a wide variety of ways (for example, to augment or adjust their existing broad self-management strategies, or to share or not share this information with others) [19,20]. Thus, fundamental questions remain about the purpose of mood monitoring interventions and how they might be adapted to be maximally useful for people with BD.

Relatively few studies have comprehensively examined how mood monitoring or ambulatory assessment interventions may influence behavior change and maintenance processes related to multiple different lifestyles, symptoms, or disease-related targets that may underlie living healthily with BD [11,18]. Ambulatory assessment influencing participants’ thoughts or behaviors is known as reactivity, and this was considered a form of confounding in early measurement studies; however, this may also be a mechanism for therapeutic change [21]. In addition to behavior change, digital tools may serve other key purposes—such as providing information for decision support (eg, is now a good time for me to move home or take on additional responsibilities?) or for emotional support (eg, is my mood where I want it to be right now?). Qualitative methods offer good potential to elucidate some of these factors [5,16]. Some protocols have built-in other features in addition to just mood monitoring or ambulatory assessment, and there is uncertainty about whether these additional features are considered valuable by people with BD. For example, in some protocols, active or passive mood data is shared with clinicians or trusted close individuals. Some protocols appear to be more connected to the functioning of formal mental health services while others appear to exist outside of psychiatric services and are solely tools to aid self-management.

This systematic review assessed the user experience of mood monitoring and ambulatory assessment protocols. Specifically, we wished to consider common themes that describe barriers and facilitators (for people with BD and for clinicians) and the intended user purpose of the mood monitoring or ambulatory assessment protocol. In this paper, we define a user as anyone who might directly use the intervention or the information that arises from it. We define participants as users with BD and clinicians in the context of the studies reviewed here. This research has not previously been synthesized and addresses key gaps in the literature around user involvement in the development of digital health interventions that are likely producing issues with implementation and engagement [3,22].

We wished to explore how mood monitoring and ambulatory assessment interventions impact the lives of people with BD both positively and negatively. Considering the frequent cessation of use of mood monitoring and ambulatory assessment interventions, we also aimed to explore under what circumstances the use of mood monitoring or ambulatory assessment interventions is maintained. We explored certain overarching concepts that had particular salience to people with BD, for example, sharing with others [20]. We assessed practical ways of addressing these barriers to use by considering desired features. We build on these results to make a series of recommendations to potentially maximize usability and acceptability in future protocols.

## Methods

### Overview

We used a methodology based on the Cochrane Handbook for Systematic Reviews of Interventions and completed a PRISMA (Preferred Reporting Items for Systematic Reviews and Meta-Analysis) checklist (see Checklist 1). The study was preregistered with the International Prospective Register of Systematic Reviews (PROSPERO CRD42023396473 [23]).

### Inclusion Criteria

We included studies if they met the following criteria: qualitative studies exploring participant or clinician perspective of self-monitoring, EMA, or repeated symptom assessment in people with BD. This included actual use and hypothetical use—we included hypothetical use as we felt that hypothetical purpose may capture early user design input that would not be captured by already developed protocols. This drew from the theory of planned behavior models capturing expectations and intentions in addition to pragmatic use—which can sometimes reveal that the reality does not meet these expectations [24].

The studies could be published in any language and could be digital or nondigital, although we acknowledged that the majority of studies would use digital technologies. We included passive ambulatory assessment studies as these have been suggested by people with BD to improve acceptability [20]. We searched the gray literature (eg, conference abstracts, dissertations, policy literature, reports via ProQuest, and Google Scholar—full details below) for unpublished studies that were eligible for inclusion. There was not, however, any gray literature that was not duplicated data that met the inclusion criteria. Studies including adults and children were included. Studies with mixed diagnostic samples were included if they also included people with BD.

Original qualitative studies, studies involving secondary analysis of qualitative data, or qualitative studies that were part of a mixed methods study (eg, the study also had a quantitative component, but the major component was qualitative, and a qualitative methodology was described) were included. Studies needed to include a substantial amount of qualitative methods, including interviews, observations, and open-ended evaluation forms. Free textboxes on evaluation forms were included if there was richness in the data provided (ie, sufficient quotes to support the analysis). Studies also needed to include a sufficient level of qualitative analysis. There were no age limits and no date limits. No other exclusion criteria were applied.

### Search Strategy and Selection Criteria

The complete search strategy is listed in the Information Section S1 in Multimedia Appendix 1. We searched Ovid MEDLINE, EMBASE, PsychINFO, SCOPUS, IEE Xplore, ProQuest SciTech Collection, ProQuest Dissertations and Theses Global, and Google Scholar using the search terms. The initial search was conducted on March 03, 2023. The range of dates was from inception to March 03, 2023. An updated search was conducted on October 28, 2024. Search results were exported for appraisal and stored on Rayyan [25,26].

### Data Screening

Titles and abstracts in the original search were appraised by independent screeners (LAW, M Majid, GM, GS, DP, and RP), and any disagreements were discussed and a consensus arrived upon, with adjudication by another independent screener if required. We acquired the full text of any potentially relevant papers, and if we were unable to source the full text of the study, we contacted the corresponding author to request the paper. To determine if potentially relevant studies met the inclusion criteria, the full text of all studies was reviewed separately by 2 authors, again with discussion and consensus with a third reviewer if necessary. All papers for inclusion were reference checked along with relevant systematic reviews [27-36]. Key authors were also emailed to see if the inclusion of any ongoing unpublished studies could be included.

### Data Extraction

LAW extracted data (first- and second-order constructs) using a data extraction form developed by LAW and SP. First-order constructs were direct participant quotes reported in the papers. Second-order constructs were the authors’ thematic interpretation of the results. The form was piloted on 3 papers initially, and adjustments were made. SP completed data extraction for 30% (6/20) of the papers to confirm congruences. Irregularities in the data extraction were discussed, and any discrepancy was resolved with discussion between reviewers.

### Assessment of Study Bias

The Critical Appraisal Skills Program Checklist [37] for assessing the quality in qualitative studies was used for each study. Risk of bias was assessed by 2 independent reviewers (LAW and SP), and any disagreement was resolved via discussion. We did not exclude studies of low quality as we wished to include papers containing depth in data collection and analysis, acknowledging that this might provide valuable information regarding user experience. We included papers that collected data through individual interviews, semistructured interviews, exit questionnaires, and focus groups. We included papers that collected data through open-response text if there was richness in the data provided.

### Synthesis of Results

A meta-synthesis approach was used to analyze the data. First-order constructs were defined as direct participant quotes reported in the studies [38]. Second-order constructs were defined as the authors’ interpretations of participants’ quotes expressed as themes, extracted from both the results and discussion sections of papers in order to capture all of the constructs [38]. Third-order constructs refer to synthesized constructs that emerge from the analysis of the first- and second-order constructs [38,39].

We used the Noblit and Hare [40] guidelines for meta-ethnography to conduct the analysis. Noblit and Hare [40] proposed 3 ways in which the synthesis of data can be achieved. The first is through reciprocal translation if the data is directly comparable. Second, if the data are in opposition, use refutational translation. Third, an integrating scheme can be produced that makes sense of the parts—a “line of argument” that uses both similarities and differences across the studies. This assessment of the included studies showed consistent themes in addition to apparent contradictions in the user experience of ambulatory assessment or mood monitoring protocols. Due to this, we used the line of argument approach to make sense of apparent contradictions in the data to then integrate the emergent concepts into a framework of user experience. This was guided by transparency, salience, and coherence with user (MM) and clinician (LAW and RM) experience. The paper is also underpinned by theories of self-management and behavior change; for example, the self-regulation and common sense model [41], social cognitive theory [42], the theory of planned behavior [24], the transtheoretical and stages of change model [43], and the technology acceptance model [44]. These models provide a theoretical framework to interpret our findings and to improve acceptability of these interventions for individuals to use for themselves as they wish.

Papers were read and reread by LAW and SP. First- and second-order constructs were extracted and managed using Microsoft Excel. Any disagreements were discussed, and consensus was agreed on. Constructs were reviewed to assess how the themes juxtaposed and compared across papers. Reviewers independently reviewed the second-order constructs, compiling third-order constructs that summarized and encapsulated the various themes across the studies using NVivo (Lumivero). Mutual discussion then refined these constructs until a consensual understanding was reached. Our results were checked for coherence by someone with lived experience of BD type 1 and over a decade of experience in patient and public involvement activity.

## Results

### Overview

**Figure 1.**
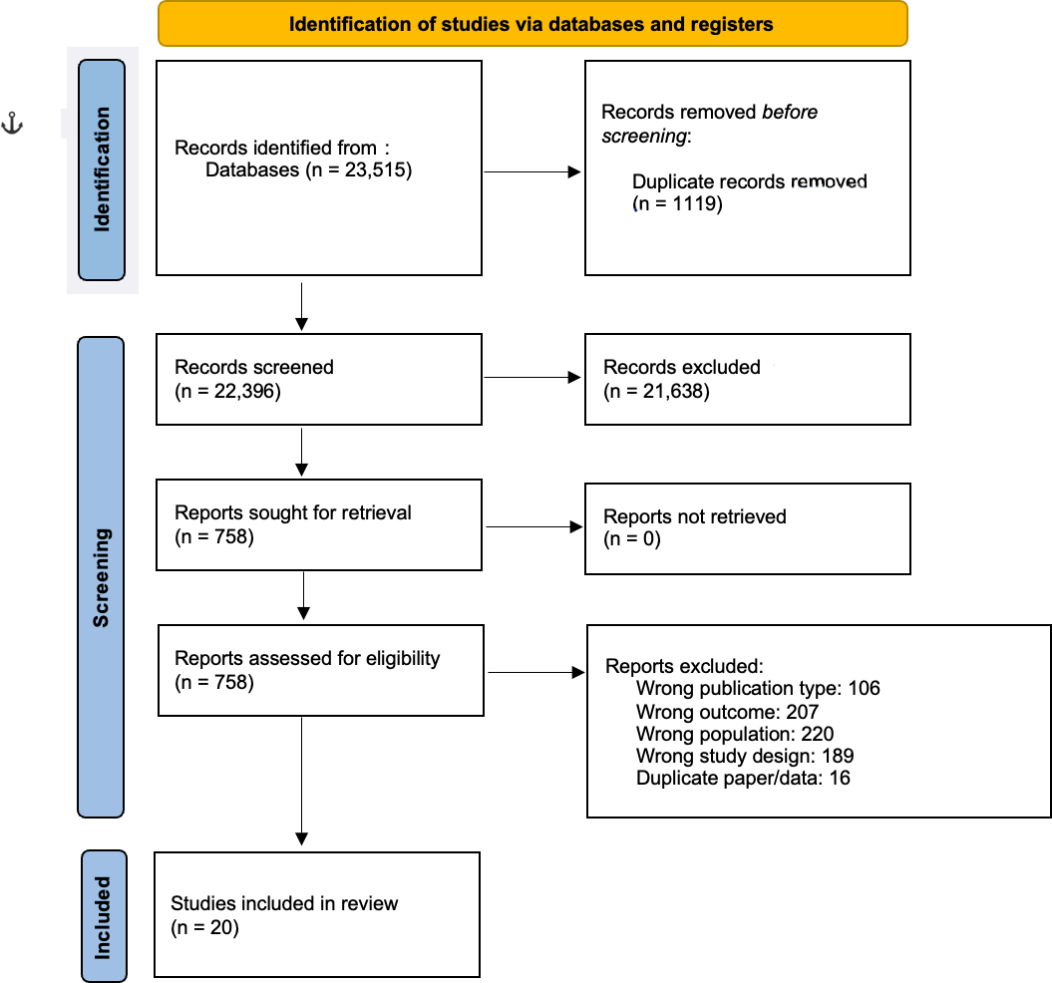
PRISMA (Preferred Reporting Items for Systematic Reviews and Meta-Analyses) flow diagram of included studies.

The search identified 23,515 papers. No studies that were not in English were found to meet the inclusion criteria. Following title and abstract screening, 21,638 were excluded, resulting in a total of 758 papers being reviewed in full. A total of 20 papers met the eligibility criteria and were included in the meta-synthesis. The other 21,638 were excluded as per Figure 1 (PRISMA flow diagram). The 20 included studies included 2365 participants. Tables S1 and S2 in Multimedia Appendix 2 display detailed characteristics of the studies and the ambulatory assessment protocols used. Table 1 reports the Critical Appraisal Skills Programme (CASP) risk of bias assessments—there were no low-quality studies included in the review.

**Table 1.** Examples of first- and second-order constructs and synthesized themes.

First-order construct	Second-order constructs	Subtheme	Third-order construct: synthesis of main findings into an explanatory framework
“Yes, yes. Maybe I’m just worried that filling in the booklet triggers something inside me. It sounds weird, . . . I know, being fixated on . . . on my depression. By writing down how depressed I feel every time, I get preoccupied with all these other feelings, what else do I feel besides depressed? You know? And maybe, I realize that I’m strongly opposed to it. Maybe I really don’t want to know about it in the end! By knowing about it and listening to it, it only feels worse!’” [45]	The patients’ reactions to the confrontation that they experienced in connection with the Life Chart Method (LCM) varied widely and appeared to relate to their individual characteristics and coping styles. A total of 6 of the 14 participants indicated that the confrontation with their limitations made them feel worse and increased resistance in using the LCM [45].	Worsens mood or anxiety.	Adverse effects: some participants report a worsening of mood and anxiety when mood monitoring and consideration of individual coping style may predict who is more likely to experience this worsening of mood.
“It came to the point where the Mood Monitoring was my least favorite [feature] because it just was so redundant with the same questions.” [46]	A few participants felt the Mood Monitoring component of the app was repetitive. This participant went on to suggest that changing the order of the questions daily could help to minimize the repetitive nature of this feature. As the questions are based on clinical diagnostic questionnaires (eg, PHQ-9^a^ and GAD-7^b^), changing the content or ordering of the questions may affect the previously determined reliability and validity of those questionnaires; regardless, it is worthwhile to note that the reaction to the repetitiveness is strong enough that it may discourage participant use of the feature entirely [46].	Repetitive nature of mood monitoring.	Barriers to mood monitoring: mood monitoring can be repetitive and consideration should be given to more acceptable measures of daily mood.
“I think it encourages you to do more exercise if you come to the end of the day and you have a choice of walking to the pub or driving I’m more likely to walk” [5].	In over half of participants, mood and activity monitoring was associated with a change in behavior. The majority of change related to activity, with most finding that they had increased their exercise levels as a result of taking part in the study [5].	Mood monitoring prompted behavior change.	Facilitators to mood monitoring: mood monitoring prompts behavior change and adjusted self-management strategies in many individuals.
“But what really allowed me to move and carry on from my episode was doing a lot of reflection and insight into myself. Insight and reflection I think were huge.” [47]	Participants described the importance of learning to pay close attention to their moods and activities, in order to judge when to make changes. Understanding their unique behavioral patterns and warning signs required self-awareness and was more common among individuals who had lived with BD^c^ longer than those more recently diagnosed [47].	Mood monitoring improved insight.	Purpose of mood monitoring: self-monitoring leading to improved self-management appears to be a complex reflective process revolving around gaining insight into the illness and potential trigger factors.
“This [feature] would need to be optional; I’d just feel like Big Brother was watching me” [46]	Some participants had hesitations with the provider dashboard due to concerns about privacy or provider interest and use. One participant felt the dashboard was too invasive [46].	Sharing with clinician feels invasive	Sharing with others (negative): automatic sharing of illness data with clinicians can feel invasive and participants expressed a desire for control over what and when to share.
“There was a lot of good information in there.to be more reflective of what’s going on. and to involve people more directly, specifically my daughter” [18]	They also noted that the intervention provided a means to share information with family members about BD and engage their support network to assist them with volitional processes such as planning and monitoring [18].	Sharing helps with planning and monitoring	Sharing with others (positive): some participants reported that sharing with their family, friends or support network helped them manage their BD. However, many participants did not find sharing useful.
“If you look at the results, it really is so hard to interpret them. It’s still much more complicated than you had hoped beforehand. On the one hand, it’s a lot of data and I like graphs and such, I think they’re nice, you have a sort of overview, and well, about activities and such, it is solid. But what comes out as predictors disappoints me. Such that I think: it’s not so unequivocal or it’s not so easy to predict. Especially for people who are so instable in their mood, then the story gets even more unclear.” [17]	Patients and clinicians further emphasized that clinicians should learn how to interpret ESM^d^ feedback and should believe in the potential of ESM [17].	Clinician barriers: difficult to interpret results.	Clinician barriers and concerns: some clinicians may struggle to interpret the results. Onboarding before use or improvements in predictive accuracy and performance may improve interpretability.
“You get to the point much more easily, and can give better targeted lifestyle advice. If they haven’t already developed those insights themselves. That is what I believe to be the advantage of self-monitoring and assignments you can do at home, outside our conversations here in the clinic: that you can adapt your own behavior and make healthy choices, so that is a nice side effect of this study, I think.” [17]	More than half of the patients made concrete behavioral changes to influence mood and symptoms (eg, becoming more active when feeling depressed, or slowing down when experiencing [hypo]mania). Clinicians confirmed this observation [17].	Clinician facilitators: prompts behavior change.	Clinician facilitators and suggestions: clinicians observed behavioral change as a result of mood monitoring and subsequent conversations between clinician and participant.

a PHQ-9: Patient Health Questionnaire-9.

b GAD-7: General Anxiety Disorder-7.

c BD: bipolar disorder.

d ESM: experience sampling method.

In total, 20 studies used 12 different ambulatory assessment protocols. The ambulatory assessment protocols were: RoQua [17], NIMH Life Chart Methodology—prospective [45,48], Livewell [11,18,49], True Colors [5,7], the QoL tool [1], Life Goals [46], C.A.L.M [50], Structured Group Psychosocial Intervention [51], Living With Bipolar [52], Personal Health Record [53], and Weekly Telephone Mood Monitoring [54]. A total of 5 studies used an open inquiry into current mood tracking practices [2,47,55-57]. In addition, 2 studies explored hypothetical use of an ambulatory assessment protocol [52,58] with the remaining 18 studies assessing actual use. The participants in all studies were reported as having a diagnosis of BD (not clarified if primary or not), apart from Bos et al [58], which was a mixed sample (see Table S1 in Multimedia Appendix 2). All studies consisted of adult participants (>16) apart from Sharma et al [50], which used a sample of young people (aged 16‐25 y). All studies explored the user experience of ambulatory assessment or mood tracking protocols, or used implementation or co-production frameworks to try to improve the usability and acceptability of the protocol. All studies apart from 3 (Suto et al [47], Murray et al [55], and van der Watt et al [54]) used digital ambulatory assessment protocols.

The synthesis revealed 9 overarching concepts: adverse effects, barriers to mood monitoring, facilitators to mood monitoring, purpose of mood monitoring, sharing with others (negative), sharing with others (positive), clinician barriers or concerns, clinician facilitators or suggestions, and desired features. These can be summarized as barriers and facilitators to mood monitoring for both people with BD and for clinicians—and we focused on certain overarching concepts that seemed to have particular salience to people with BD, for example, sharing with others [20]. Table 1 demonstrates examples of first- and second-order constructs and synthesized themes and provides an illustration of the analysis.

### Adverse Effects

A total of 10 studies reported on adverse effects (see Table S2 in Multimedia Appendix 1), and we reported 7 subthemes. Participants in 7 studies reported feeling that mood monitoring was worsening their mood or anxiety. This worsening was often in response to perceived negative feedback delivered by the ambulatory assessment protocol or by repeatedly inputting their mood was poor or worsening. This realization of where their mood was appeared to cause some individuals distress. Becoming fixated on their mood being low and the presence of their depression undermined their expectations of recovery in a way that was distressing. Another subtheme was the burden of mood monitoring—certain users reported feeling that managing their health with the ambulatory assessment was arduous, time-consuming, and burdensome. A few users felt that it required a high level of self-expertise that was challenging for someone in the early phases of their BD. Relatedly, several users found the mood monitoring confronting, with some reporting this was a necessary part of managing their illness, with others finding it distressing to feel like they were managing their disorder continuously. For some, the process was an unhelpful reminder of a mental illness that for many was chronic and unstable. Various users reported that their daily focused mood monitoring became compulsive and difficult to step away from. Other subthemes reported were feeling that ambulatory assessment limited rather than enhanced their ability to self-manage (in part due to feeling that the protocol did not sufficiently capture their desires, needs, and possibilities) and fears of a loss of autonomy, particularly when other people were involved in the ambulatory assessment process.

### Barriers to Mood Monitoring

A total of 13 studies reported on barriers to mood monitoring (see Table S3 in Multimedia Appendix 1). A user reported that active ambulatory assessment notifications and prompts often arrived at an inconvenient time. Many users felt that repeatedly reporting their mood was monotonous and repetitive, especially if the EMA was repeatedly providing negative feedback about an issue they had not successfully addressed and that this decreased motivation. Similarly, many users discussed the large amount of time, energy, and commitment required to use the protocols particularly over the medium and long term. Some individuals felt that the questions asked by mood monitoring were not particularly relevant to them, were limited, and did not capture the range of their mental experience. Others disliked the impersonal nature of the repeated questions. Participants struggled to complete the measures both when well—due to a lack of interest and urgency in managing their BD—but also when unwell, as a lack of insight frequently removed motivation to complete the measures when manic. For some, the mood monitoring just confirmed what they already knew, adding nothing new.

For others, the data was difficult to interpret and then use for their own self-management, particularly the large volume of the data. Some participants found the graphs difficult to understand and wanted feedback or interpretation of what their data represents or means for their BD. Ambulatory assessment protocols can be an unreliable judge of mood with some participants who reported difficulties in judging their mood on a simple scale; for example, some struggled with connecting to their feelings and summarizing this numerically. A few users reported their mood as the same consistently for long periods of time, and this caused them concern about the accuracy of the protocol to distinguish between mood states. Indeed, others mentioned that using the mood monitoring tool at the right stage of their BD was key. Users reported time since diagnosis, their knowledge of the disorder, their own degree of insight or acceptance, and sensitivity to the implications of the diagnosis for their life as key factors when choosing the right time to commence mood monitoring.

Data security concerns were a large consideration for many, with some expressing a desire for safeguards to ensure the data was kept private, anonymous, and not shared with external companies or organizations not involved in the direct care of the individual. Furthermore, use of the protocol was sometimes interrupted by technical issues prompting frustration and irritation. Some protocols were felt to be too cumbersome with multiple technical obstacles to overcome before use—specifically quickly logging in to report their daily mood. Users reported wearable failure to capture their activities and subsequent concerns about data accuracy, as well as poor phone battery life on the supplied equipment.

### Facilitators to Mood Monitoring

Passive ambulatory assessment was felt to improve the overall experience of mood monitoring and to compensate for some of the loss of insight when unwell that made mood appraisal a challenge (see Table S4 in Multimedia Appendix 1). Automatic, passive monitoring was perceived as advantageous as it required less manual input and relied less on remembering to input the data. Many users were highly receptive to integrated systems using wearables to complement data collection. Furthermore, the wearables were well tolerated by many. In contrast to other users, some felt that once-daily mood monitoring was not inconvenient and did not take up an excessive amount of time.

Many of these mood monitoring protocols were a natural adaptation for users as they were already monitoring their mood using a variety of different techniques, for example, using paper and pen-based techniques, or informally keeping track of personal indicators mentally. Some used social feedback and communication with those around them to supplement their internal sense of what their mood was. Participants felt that the ambulatory assessment protocols prompted behavior change—specifically in terms of routine keeping, taking medication regularly, staying active and doing more exercise, prioritizing their sleep, and goal setting or monitoring across a range of behavioral targets.

### Desired Features

Users reported a wide variety of desired features for consideration of inclusion into future EMA protocols (see Table S5 in Multimedia Appendix 1). Key factors were ease of use—with simple, quick, and user-friendly methods of logging their mood. Some users had a preference for simple, functional, and minimalist layouts that encouraged regular use and quick reporting of mood. A participant suggested the removal of a separate log in every time the app is accessed to reduce the technological barriers to inputting mood. These ideas tie in closely with the technology acceptance model [44].

Users expressed a desire for control over many aspects of the protocols’ functionality—including what to track, what, when, and who they share data with, whether they use the ambulatory assessment or not, and whether they receive notifications or not. Fundamentally, a desire was expressed to opt in to various features (such as what and when to track and who to share this with) rather than these features requiring opting out. Some users did not want any link with their social media accounts.

While participants wished to be able to control the frequency of notifications they receive, many considered the notification reminders to complete the ambulatory assessment essential and useful. Furthermore, medication reminders were perceived as useful by many. A preference was expressed for digital methods as these were reported to be more accessible and interactive, but this came with an awareness of data transparency—guarantees of privacy and the data not being accessed by external organizations or companies.

Some users wished to incorporate additional features into the protocol—these included: a diary, financial tracking, uploading of audio and video recordings or music, text, or photos, integration with other aspects of physical health, and crisis or well-being plans. The incorporation of a crisis plan was mentioned multiple times by multiple users. Some users wished to track metrics beyond just mood, including: energy, alcohol, exercise, diet, substance use, caffeine use, pain, libido, suicidal ideation, chores, pet care, leisure activities, cooking, and hormone tracking. Considering the difficulty of use of some protocols and difficulty of interpretation of the data, some participants wished to receive some additional coaching, support, or onboarding to best use the protocol.

There was a large and explicit emphasis on flexibility, customization, contextualization, and personalization. Some users felt that the ambulatory assessment protocol they followed was too generic and rudimentary to allow the degree of personalization (personalization subtheme found in 10 studies) they desired for accurate insights into their BD (eg, the questions used to assess their mood). Users felt that personalization improved their motivation to engage with the protocol and their perceived value from it. Many wished the feedback they received to be more personalized. Examples of personalization included personalized wellness anchors or early warning signs, personalized crisis plans, and the monitoring factors that participants felt were particularly relevant to them at particular mood states.

In addition to this, participants expressed a desire to receive positive messages and feedback from the protocol. Participants also wished to track positive developments in their life, such as tracking strengths and improvements. A participant felt that this helped in reclaiming their identity beyond the sick role of BD. Other users felt that tracking the positives further motivated them to push themselves to socialize and exercise more, which had additional benefits on their mood.

Finally, the scientific quality of the protocol was important to some, with some users focusing on whether the protocol was useful or effective for people with BD.

### Purpose of Mood Monitoring

Participants used the information gained from the mood monitoring protocols in a wide variety of ways for a wide variety of purposes, but often this purpose was not built into the protocol (see Table S6 in Multimedia Appendix 1).

Principally, users felt that the ambulatory assessment improved their insight and self-awareness—for example, understanding patterns and changes of their mood, prompting reflection, developing an understanding of potential triggers, and considering possible ameliorative factors. In some individuals, this understanding prompted conversations about their experiences with trusted individuals, for example, friends, family, and carers. These conversations seemed to further improve insight, providing a framework for discussions around the management of their BD. Users appeared to use the information to implement self-management strategies that varied widely. Strategies included scaling back activities to reduce external sources of stress, adjusting or initiating medication, prioritizing sleep, consulting their relapse prevention, or crisis, or wellness plan, and communicating clearly with individuals close to them. The protocol specifically enabled some participants to stick to a regular structured routine. Users placed particular emphasis on the role of monitoring sleep and medication adherence. Mood monitoring with self-reflection and subsequent trialing of different coping strategies appears to be an iterative process that mood monitoring can contribute to and might be difficult to achieve without.

Objectively judging mood seemed key to then promoting insight and implementing key self-management strategies. Some participants reported greater understanding and awareness of early warning signs subsequent to objective evidence of their mood, allowing them to make changes to their lifestyle and ultimately prevent or plan for relapse. This information was used to aid and monitor treatments—to assess possible benefits from both medication changes (sharing this information with their doctor) and changes they have made to their lifestyles. Many individuals used the protocol to motivate themselves to continue to manage and improve their health. This seemed particularly relevant if the participant felt that things were generally improving. Some felt that engaging with mood monitoring improved their sense of autonomy and allowed them a greater degree of responsibility in managing their BD. In addition to objectively judging their mood, contemporaneously many users wanted to be able to retrospectively assess a record of their mood over the long term to recognize progress and additional patterns to short term monitoring, for example, charting data to assess for seasonal variations. Certain users found the sense of objectivity here reassuring.

A combination of many of these different purposes and uses of the data was felt to prevent relapse. Users wished to use the protocols in different ways at different times—contemporaneously, some wished to only use it occasionally, while others wished to use it all of the time. There were times when mood tracking was particularly important and times when it appeared less important. Some participants reported using external tracking when they needed extra support—for example, when their thoughts felt scrambled or their mind felt too full—sensations they identified when their mood was hypomanic or manic.

### Sharing With Others (Negative)

The positives and negatives of sharing the protocol data with others were a particularly prominently discussed feature and one which we explore separately here (see Table S7 in Multimedia Appendix 1). Whether or not users wished to share any of their data varied widely, and some views were difficult to combine into a coherent unifying thesis.

Several users did not want to share the data with their families for fear of burdening them, while others felt that their families would not understand. Sharing was felt to be of limited helpfulness with many recipients of the data or information finding it hard to relate the data to the individual and some not taking the data seriously. A few recipients reported sharing with some individuals and feeling that this caused the recipient worry, anxiety, and a perceived overreaction. Various users were concerned about medicalizing the relationship, with their friends or family adopting the role of a care provider. Many regarded the data as a sort of private diary, and it did not feel appropriate to share that.

Furthermore, users seemed to prefer opting in to sharing (in both personal relationships and with healthcare practitioners but for different reasons), and retrospective sharing was often preferred when considering any involvement with their health care practitioner. Users spoke of the importance of retaining control over their data. Users reported separate concerns that sharing the data with their health care practitioner felt invasive, and worries were expressed about privacy—users worried about being judged or control being exerted on them by mental health services. Some expressed fear of mental health services—a principal worry was of their data being negatively interpreted with consequences such as involuntary hospital admission. Participants felt that the context of their data was important and that this may not be considered by their clinician when reviewing the data leading to potential perceived overreactions. Others felt that their health care practitioner probably would not look at their data, thus negating any purpose of sharing.

### Sharing With Others (Positive)

Participants shared with their friends and family to improve understanding and communication—for example, appreciating the relationship between mood and behavior, particularly in the context of the relationship. Some people with BD felt it was easier to share their data than describe how they felt to others. Some reported analyzing the data with their trusted individuals. Often these trusted individuals helped with monitoring their mood, reflection, and planning in the event of relapse.

Some participants wished to share their data with their clinician to efficiently convey a lot of information to improve their clinical care—particularly in monitoring treatment, for example, medication response over time. The data also facilitated detailed conversations about their illness such as psychosocial factors that may be affecting mood, relapse prodromes, early warning signs, and identification of unusual mood states, for example, mixed episodes. Some users were happy for their clinician to automatically receive their mood data, with the users’ consent, although it was unclear if this referred to real-time mood data or retrospective mood charting.

### Clinician Barriers and Concerns

Clinicians had several concerns about the implementation of ambulatory assessment in clinical practice (see Table S8 in Multimedia Appendix 1). Some clinicians felt that people with BD could be unreliable judges of their own mood and sometimes lacked insight to self-assess mood. Concerns were raised about the burden of mood monitoring for people with BD and the amount of consideration, commitment, and time required to then devise and implement self-management strategies. Some clinicians felt that ambulatory assessment could exacerbate the low mood of their patients by highlighting periods of depression. Concerns were also raised that focusing too much on day-to-day change, rather than appreciating the bigger picture, may worsen mood.

Clinicians found it difficult to interpret some of the mood data, particularly the raw data or when there were frequent or rapid shifts in mood. Some commented that translating the data into a more usable format was key in improving the clinical use of the ambulatory assessment. Clinicians also considered that the sharing of any mood data may create risk and liability issues—increasing workloads and adding additional responsibility to their role.

### Clinician Facilitators and Suggestions

Similarly to the users, clinicians also agreed that the ambulatory assessment helped guide treatment and response to treatment (see Table S8 in Multimedia Appendix 1). Clinicians also felt that the mood data improved communication and provided a framework to discuss medication changes, psychotherapy, and general self-management of the user’s BD as part of an in-person appointment. Some clinicians appreciated that the mood monitoring did improve insight, self-awareness, and understanding of relapse triggers, and that this prompted observable behavior change. Certain clinicians felt that the advice they could give around lifestyle was considerably more targeted because of receiving the mood data.

Some clinicians suggested that personalization of the protocol was paramount to cater to the high degree of variability in people with BD. Suggestions were also made to link the ambulatory assessment to a relapse prevention plan.

## Discussion

### Principal Findings

This systematic review assessed the user experience of mood monitoring and ambulatory assessment protocols. We explored barriers and facilitators to mood monitoring for both people with BD and for clinicians. We explored certain overarching concepts that had particular salience to people with BD [20], for example, sharing with others. We assessed practical ways of addressing these barriers to use by considering desired features. We build on these results to make a series of recommendations to potentially maximize usability and acceptability in future protocols.

The themes emerging from the meta-synthesis acknowledge the variability across the user experience of ambulatory assessment protocols in people with BD. Users recorded their mood data and used this to self-manage their BD in a wide variety of ways, many of which were felt to be beneficial in terms of improving insight, assessing their mood objectively, adjusting their lifestyles, and preventing relapse. Many of these self-management techniques need not be built into the protocol, but the protocol should allow and permit personalization and flexibility, which was an overarching theme. This preference for personalization and flexibility also included control over the mood data and data security issues more broadly, in particular, what is shared with who and when. Some users perceived the protocols negatively, noting adverse effects and offering large scope for improvement. Principally, users expressed that these intervention protocols should sit outside of conventional mental health services, with control over users’ mood retained principally by them with clinician involvement if desired. Clinician-perceived facilitators and barriers were largely shared by those with BD. For example, the following subthemes were shared: worsens patient mood, burden of mood monitoring, difficult to interpret results, helps guide treatment and medication response, prompts behavior change, and personalization. The following subthemes were only raised by clinicians: concern about risk and liability issues, self-appraisal of mood can be unreliable. There did not appear to be any significant disagreements between the views of clinicians and those of people with BD, and it is natural that clinicians are more liability-focused than people with BD.

### Facilitators

People with BD expressed many facilitators to continued mood monitoring. Many people were already tracking their mood, and these protocols provided a formal framework to continue to mood monitor. There was a clear perception that the ambulatory assessment helped many users to prevent relapse, improve understanding and insight, and to help them to self-manage their BD. This self-management was often separate and distinct from their involvement with formal mental health services (for example, the self-regulation model [41]). Users expressed a preference for consciously involving mental health services when they wished to (with many not wishing to)— this sharing was often in retrospect rather than in real time. The ambulatory assessment served different purposes in different contexts, and some of these were incompatible. Examples of these are: (1) self-management or monitoring without any involvement of mental health services, (2) mood monitoring occasionally shared with mental health services, and (3) mood monitoring for mental health services.

### Purpose

People with BD used the data the protocols provided to them in several ways – these were highly personalized and tailored to what appeared to work best for them. In many ways, the mood monitoring provided a platform for people to then interpret their own mood data—devising highly personal ways of self-managing their BD over time. This appeared to be an iterative process with self-management styles adapting and changing as the users became more familiar with the protocol and as they gained insight into their condition. Clinicians felt that when the data was richer, they were able to make more targeted lifestyle recommendations to protocol users. This also appeared to be true for people with BD when interpreting their own data—data that was more personalized and more detailed in terms of information was valued more highly and helped individuals to make key decisions around their self-management (for example, the social cognitive theory model [42]).

### Personalization

Future ambulatory assessment protocols should consider incorporating personalization and customization—both in terms of the data the protocol collects, but also in terms of how this is fed back to the user. This may require onboarding and support for the user to comprehend how to maximally use the system. Personalized approaches appeared to improve motivation and perceived value, and this may decrease attrition and improve adherence to the protocols, which is often of concern to mental health apps in general [28], not just for those specifically aimed at BD [59]. User involvement in many self-monitoring apps is often unclear and likely low in many apps [22]—this frequent failure to consider the needs and goals of people with BD will likely contribute to poor engagement [3]. Future protocols should allow users to opt into the different facets of data collection—be this through active or passive ambulatory assessment—and opt into various methods of personalized feedback that the user may receive. We report the importance of data security with data sharing, something that a user opts into rather than occurring automatically and data not shared with external companies.

Personalization, however, might be expensive, technologically demanding, and potentially less reliable in certain respects than a more uniform technology. It might also involve the use of artificial intelligence, which people with BD and clinicians might mistrust [60,61]. There are different aspects of personalization to consider, and it is possible that the technology might be less personalized, but the protocol in relation to how the information is used might be personalized. These include things such as the functionality about how a piece of data is used, the choice to select what could be viewed, how data is visualized, the threshold for alerts. This could be a key method of incorporating personalization in a resource-constrained health care setting or one that has limited finance available for end-user involvement in the development of digital mental health technology. If the technology is more personalized, then that might include some of the functions being switched off for some people. Personalization may be key in driving success via the technology acceptance model—if the perception of the intervention is of usefulness, ease of use, and applicability to their life, they are more likely to use it.

### Context in Previous Research

This accords with similar research assessing the user experience of other digital health interventions for individuals with depression, anxiety [38], somatization disorders [62], and patients, the public, and health professionals more broadly [63]. Personalization was found to affect the overall perception of usage, with high levels of consideration for individual preferences and circumstance being associated with positive perceptions [62]. A need to experience a sense of “self” in the digital health intervention was an overarching factor in both the usage of intervention [38] and recruitment to and overall engagement with the intervention [63]. This review highlights a similar requirement for the sensitization of content to people with BD using ambulatory assessment or mood monitoring interventions.

### Future Recommendations

Future ambulatory assessment protocols should carefully consider the form of repeated measurement used. Many participants felt that the questions asked of them did not reflect what was most important to them. Participants found many of the questionnaires repetitive, and this decreased motivation to complete the ambulatory assessment protocol. Various participants reported stopping reporting their mood with any real effort and started to just click through it. Future ambulatory assessment protocols should consider alternative measures of mood to existing validated self-report questionnaires such as visual analog scales or more usable and acceptable measures that decrease any potential participant burden. Notifications to complete the ambulatory assessment were at times perceived as excessive and at other times perceived as essential and necessary to individuals getting the most out of the mood monitoring. Customization of these notifications is thus important for any future protocols. This raises some points around the user experience of the protocols—these are likely to be protocol-specific and will require iterative testing by people with lived experience of BD. As we demonstrated here, some individuals with BD have clear preferences around the interface and visual display, with one user commenting, “I would like to see a kind of scroll function that you zoom in or out on the graphic of the [mood chart]; I think that would be absolutely fantastic.”

Some users considered the scientific validity of the protocol important and fundamentally asked the question—is this effective and will this work for me? This emphasizes the importance of both scientific performance and clinician or institutional approval but also of user reviews of tools. The performance of many ambulatory assessment protocols is unclear [28], and there was a large variation in the diversity of protocols, which may prevent inference of performance from other evaluated protocols. Many of the purposes of the ambulatory assessment or mood monitoring seemed to depend on good performance of the protocol—particularly to accurately judge mood state that was the core functionality that led to improved insight, understanding, and awareness of mood and subsequent implementation of self-management strategies. Future ambulatory assessment protocols should evaluate and consider performance in their development. Other studies of both people with the relevant conditions and clinicians [64-66] have confirmed the need for some degree of validity and reliability as well as precision in the measure.

Furthermore, preparation and onboarding before engaging with mood monitoring seemed important with many participants expressing a need for assistance to aid interpretation of the data. This could take the form of onboarding or flexible coaching alongside the use of the protocol. This is a frequent request for technology related to health—many individuals do not want to misunderstand their health data because of its potential serious consequences [67]. Onboarding may also help with those who find the use of technology increases awareness of low mood, for example, by teaching strategies for dealing with this awareness, for example, mindfulness, cognitive restructuring, or distraction techniques. Some users highlighted that they felt they needed to use the protocol at a particular stage of their BD—for example, when they had a degree of insight already that they could then build on, rather than the monitoring becoming too overwhelming, for example, at an early stage of one’s BD (eg, the transtheoretical or stages of change model [43]).

For some topics considered in this review, there was a diversity of opinions, some of which were contradictory. Some users wished for a protocol that was minimalist with few technological barriers to inputting their mood. Others were concerned about data security and wished for additional restrictions to access their data. Some users expressed a desire for multiple additional features that would create a more complex and multifaceted app—for example, also tracking a range of additional lifestyle and physical health features. Incorporating this diversity of views and considering the multiple needs of a diverse group of individuals (themselves bringing a huge spectrum of experience with the nature of their BD and how it affects them) will be a challenge for future protocols. There are also potential issues around digital literacy and access to technology in the context of potentially disabling symptoms that may need to be considered moving forward, and here we discuss the potential need for onboarding to use these tools.

Quantitative studies have failed to demonstrate robust evidence of adverse effects [29]. Here, some participants report a worsening of their mood and anxiety due to mood tracking, while others reported beneficial effects in terms of relapse prevention and improved understanding. It is likely that only a proportion of individuals experience this adverse effect—possibly due to improved insight when experiencing low mood further exacerbating depression. It is also possible that while some participants feel that it worsens their mood, this does not produce clear effects when depression is measured quantitatively, or that these quantitative measurements do not capture this nuance. Furthermore, these studies may not have been powered to assess an adverse effect affecting the minority of participants. This study builds on existing research by summarizing the qualitative reports of adverse effects in the absence of conclusive quantitative evidence [28,29].

It is difficult to strike a balance between the potential distress caused by this insight and the potential therapeutic value of improved self-awareness and insight. Some adverse effects could likely be managed by adjustment of the protocol. Some users considered the experience too confronting, and for them, it was a too frequent and unhelpful reminder of having a mental illness. Predominantly using passive ambulatory assessments rather than active ambulatory assessments may decrease the regular contact with the protocol and decrease the amount of time users are actively required to engage with the protocol, and this was preferred by many participants. This may decrease some of these negative reminders of their illness and potentially decrease the prominent feeling of mood monitoring as a burden that many users reported but would require further user assessment and patient and public involvement (PPI) involvement. It is likely that maximizing the therapeutic value of the tool will be pertinent to mood monitoring in other mental health conditions, for example, depression—where similar adverse events are also reported [68]. While there are very few reviews examining the qualitative user experience of mood tracking, a review examining depression is currently in press [68].

### Strengths and Limitations

This is the first systematic review, to our knowledge, that has assessed the user experience of mood monitoring and ambulatory assessment protocols. Input from people with BD is vital in ensuring the successful implementation of ambulatory assessment and mood tracking technologies [69] and our results were checked for coherence by someone with lived experience of BD. We used a robust methodology in line with Cochrane review standards to include as many papers as possible. The studies were relevant, of high quality, and only 2 studies examined hypothetical use, with the remaining studies assessing actual use. The results of these 2 studies did not appear to be any different compared to the other 18 studies, and this may suggest that the actual use does not differ much from the expected use.

A common limitation of reviews is the combining of multiple heterogeneous studies. First, some of the user experiences we describe here relate to specific, more restrictive or unfocused protocols and not others. Second, some of the subthemes in our results relate to specific studies and protocols that may be problematic in some salient ways, or alternatively may be power-related. This review is the comparison of a wide range of ambulatory assessment protocols incorporating a variety of features. Nonetheless, all these included frequent mood assessment at the core of the intervention. Despite the range of protocols, many themes, such as customization, were shared across multiple studies despite any protocol differences supporting the generalizability of the findings. One theme was only explored in a minority of papers such as clinician barriers and facilitators that principally relied on the results from only 3 papers. This was an exception, however, with the remaining themes having sufficient data from all papers. All studies were English-language papers, and given the major sources of technology production in China, India, and Japan, it is possible that we may have missed some relevant non-English speaking publications, although non-English language was not an exclusion criterion.

### Conclusion

To conclude, assessment of user experience of mood monitoring and ambulatory assessment protocols demonstrates the potential to adjust existing protocols to maximize usability, acceptability, user experience, engagement, and adherence. We have made multiple recommendations that future protocols should consider ranging from technological solutions to issues of performance. This research highlights the considerable variance in views and opinions from participants. Unsurprisingly, there does not appear to be one universal perspective on the components and user experience of ambulatory assessment or mood monitoring protocols. Fundamentally, users wished to retain control over their data with a high degree of emphasis on customizability and personalization. This personalization included the type of data collected, the data shared, the feedback the protocol offers, and notifications. We advise that customizability be placed central to future protocol development and incorporated using the recommendations made in this paper to maximize user engagement and successful uptake.

## Supplementary material

10.2196/71525 Multimedia Appendix 1 Additional methods and results not reported in the main text.

10.2196/71525 Multimedia Appendix 2 Characteristics of included studies and risk of bias assessments.

10.2196/71525 Checklist 1 PRISMA checklist.

## Abbreviations

BD: bipolar disorder
EMA: ecological momentary assessment
