# Supplementary material for: The User Experience of Ambulatory Assessment and Mood Monitoring in Bipolar Disorder: Systematic Review and Meta-Synthesis of Qualitative Studies
Source: J Med Internet Res. 2025 Oct 17;27:e71525. doi: 10.2196/71525 (PMC12533931; doi:10.2196/71525)
Supplement: Multimedia Appendix 1 [file jmir-v27-e71525-s001.docx]

Table S1: examples of illustrative quotes to demonstrate sub-themes and third order constructs

| **Third order construct: synthesis of main findings into an explanatory framework** | **Sub-theme** | **Illustrative first/second order construct** |
| --- | --- | --- |
| Adverse effects | Worsens mood and/or anxiety | Yes, yes. Maybe I’m just worried that filling in the booklet triggers something inside me. It sounds weird, . . . I know, being fixated on . . . on my depression. By writing down how depressed I feel every time, I get preoccupied with all these other feelings, what else do I feel besides depressed? You know? And maybe, I realize that I’m strongly opposed to it. Maybe I really don't want to know about it in the end! By knowing about it and listening to it, it only feels worse! |
|  | Unhelpful reminder of mental illness | I don't want to be reminded of being down. I'm aware I don't do it. |
|  | Decreases sense of autonomy | Whereas I always believe, you know, people really have to reach out themselves. |
|  | Limits ability to self-manage | They feel that their self-management ability is limited as opposed to enhanced by working with the LCM. |
|  | Mood monitoring feels confronting | It wasn’t the design of the instrument; no . . . it was purely and only, the confrontation. Writing down how you feel, every day. . . . I found it very confronting and sometimes it still feels that way. |
|  | Mood monitoring can become compulsive | Being involved with the disease every day by the LC can eventually become compulsive. |
|  | Mood monitoring feels like a burden | For these respondents, health management can seem like a full time job that leaves little time for anything else, and tracking provides enough organization to ‘move past sheer maintenance and into the realm of improvement’. |
| Barriers to mood monitoring | Inconvenience of active ambulatory assessment | Especially when the assessments come at an inconvenient moment. |
|  | Adverse effects | Occasionally, it’s very good to get some distance from the booklet . . . having this disease, I think . . . and try to live an ordinary life. |
|  | No additional benefit | And it mostly was a confirmation of everything I already knew. |
|  | Data security concerns | Only being stored locally on the device instead of the cloud. . . not collecting a seemingly unnecessary amount of data, not linking it to anything else that’s personally identifiable such as. . . home address/real name. |
|  | Difficulties interpreting the data | Having some kind of reassurance about that or explanation I think would have been very very useful |
|  | Difficult to track mood when unwell | I stopped because I was not feeling lekker [good] at that time. I was in low. I cut everybody out of my life. Therefore I decided to stop the study. Around that time I did not want to go to hospital. |
|  | Requires a lot of commitment and energy | It's a lot of maintaining and you're constantly working on it. |
|  | Mood monitoring feels impersonal | Others also noted a dislike for the impersonal (non-contact) nature of the study. |
|  | Lack interest in tracking when well | Patients in a euthymic phase sometimes found it difficult to maintain charting because of a loss of interest and lack of urgency. |
|  | Mood monitoring superficial and restrictive | I always hated the mood diaries, it seems too constricting to just rate my mood on a scale of 1 to 10 for one day. |
|  | Repetitive nature of mood monitoring | I got fed up after a couple weeks ‘cause [the Daily Review] was the same thing. |
|  | Mood monitoring unreliable judge of mood | I almost answer the same things everyday like you know so I don’t, so I don’t feel like it was maybe accurately measuring me well enough. |
|  | Use at the right stage of your illness | Some participants stated that the introduction of the LCM came too soon within the process of accepting a diagnosis of BD. |
| Facilitators to mood monitoring | Advantages of passive  ambulatory assessment | Nearly all respondents conveyed high receptivity to the idea of more intelligent tracking systems that passively mine behaviors, automatically detect and predict affective changes, and report feedback to the user about his or her current state, the potential onset of symptoms, and appropriate coping strategies. |
|  | Already using a variety of mood monitoring methods | The forms of paper-based tracking that participants mentioned us- ing include journals, sticky notes, charts, and calendars. Some re- spondents print out templates found online, while others create individualised scales, in which, for instance, numbers correspond to self-defined mood states. |
|  | Active ambulatory assessment not over-burdening | No, the amount of work itself was not that much. |
|  | Mood monitoring prompted behaviour change | I think it encourages you to do more exercise if you come to the end of the day and you have a choice of walking to the pub or driving I’m more likely to walk. |
|  | Wearables well tolerated | Unobtrusive – soon forgot I was wearing it. |
| Desired features | Other aspects in addition to mood monitoring | Adding tracking for energy level, alcohol use, diet, and exercise. There should be more visual things . . . like video clips. |
|  | Crisis/wellness plan | I’ve had a couple of plus two days and this, this and this is happening... It was nice to look at [my Wellness Plan] and say okay I have some things that I can do to try to bring this down and if it doesn’t go down I know that I need to make a phone call. |
|  | Data transparency | No access to the personal data by ICT-company or others who are not involved with the treatment. |
|  | Preference for digital methods | Internet is the only format: freely accessible, instant and interactive. |
|  | Effectiveness/scientific evaluation | For a user to perceive benefit the app should be helpful. |
|  | Ease of use | The app should be easy to use, simple but effective features. A health app shouldn’t try to be flashy or innovative, it should be streamlined and functional. |
|  | Full control over many aspects of the process/app | NO link with other social media to prevent uploading of personal data from the LC during an episode. |
|  | Graphical display of mood | I would like to see a kind of scroll function that you zoom in or out on the graphic of the LC; I think that would be absolutely fantastic. |
|  | Medication reminder notifications | I had a hard time remembering to take my medications and being motivated once I did forget to take them...with [LiveWell] you could at least say 100% every day on medication so that really helped. |
|  | Reminder notifications to complete ambulatory assessment | User-friendly, using LC indicators, additional options for monitoring, sending reminders every day (to be set up), giving positive feedback. |
|  | Personalisation | We personalised [the Wellness Plan], going through each mood variation level and noting what I’ve personally experienced. At first, it was really generic, so going through it and having it be like ‘if you are up a level, this is what you are going to be seeing’ . . . It’s my plan, not just a generic one. |
|  | Positive messages | I feel like the tool is good even when you’re doing well because, any kind of positive, anything that makes me feel good about myself, is always positive... That’s kind of what it feels like, that encouragement, that yes, you’re doing good, you’re doing a good job. |
|  | Support required prior to/during use | Moreover, users felt that the coaching support was motivating, useful, and helped them get more out of their app use. |
|  | Tracking additional features other than mood | participants reported that they track various other items that are relevant to their condition, including medication, side effects, and doctor’s appointments as well as personal triggers and manifestations of symptoms, such as caffeine and alcohol intake, pain levels, appetite, libido, suicidal ideation, and self-harm. |
| Purpose of app | Aid/monitor treatment | I think they’d be able to make, ... make objective decisions about medication changes . . . and frequency of interaction and yeah freq- ..., yeah, yeah, ... it’s, it’s ... having, ..., having ... a little of our data about our moods. |
|  | Improve communication | My partner and I use the graphs to look back at the prior week and attempt to identify any reasons for the changes in my mood. |
|  | Early warning signs | I’ve learned my early warning signs [and] I make minor adjustments. |
|  | Reassurance | I feel it's like having a third party to keep an eye on me! |
|  | Improves autonomy | This [referring to the BDMM Study] holds you accountable to yourself. |
|  | Improve insight | Thinking it through helped me be aware of my behaviors and my sleep patterns especially. |
|  | Monitor long term mood | Another participant noted that they appreciated the Mood Monitoring graph as it was helpful to “visualize mood trending upwards or downwards over time”. |
|  | Monitor medication adherence | LiveWell was a reminder to take my meds. If I wasn’t going to bed, I would remember to put them close to my bed. |
|  | Monitor sleep | I definitely started trying to stay within that window of for going to bed...I started noticing when I wasn’t getting the right amount of sleep or when my schedule was very off...It helps me course correct a bit faster. |
|  | Improve motivation | It made me feel really good actually, because I knew I was on the right track... yes, I suffer from bipolar, and now it’s [participant’s name] and I’m going to work, I’m functioning, full time hours, and able to handle a lot more than I used to. |
|  | Objectively judge mood | [My strongest memory was] definitely the check in and the rating of myself. That was the biggest part of the check in for me to have that time to sit down and really say like okay for the last 24 hours how was I really? I’m good now but let’s think back, or I'm not doing so well, what happened in the last 24 hours? Was it situational or was it not situational? |
|  | Relapse prevention | [My] strongest memory is pulling my mood back from that mild up. It was a big deal. I was really glad to have that phone in my hands when I realised that was happening. |
|  | Helps stick to routine | The thing that helped me the most was trying to stick to a routine... I needed more routine. |
|  | Improves self-management | If I have 2 or 3 nights with less than 6 hours of sleep, something is gonna happen so I make sure my husband is the person who takes care of the kids that night and I’ll sleep in the guest bedroom. |
|  | Periods when tracking more important | Finally, open-ended survey question responses suggest that an individual’s tracking practices can shift over time and with the severity of the condition. |
|  | Use mood monitoring in a variety of different ways | The frequency of using the LCM varied widely among the patients who completed it. Some used it occasionally; some used it on a daily basis. |
| Sharing mood-monitoring data | Negative: don’t want to burden others | Patients sometimes did not talk about the LCM because the life chart was regarded as a private diary, they did not want to burden their families, or they did not want to be perceived as ill. |
|  | Negative: others don’t understand | ... [to talk to someone] who seems to understand, since family doesn’t always understand you and the disorder ... [it is an] opportunity to change things. |
|  | Negative: don’t want to medicalise other relationships | I don’t share it with other people, no. . . . Because, um, I prefer that my mother is my mother and my brother is my brother. They are my family and not my care providers. |
|  | Negative: others overreact to the information I share with them | I find my partner worries too much about me and over reacts. |
|  | Negative: I want to maintain my privacy | Patients sometimes did not talk about the LCM because the life chart was regarded as a private diary. |
|  | Negative: clinician is not interested in the information | Respondents who encountered problems related to clinicians not accepting or valuing their self-tracked data explained that such rejections leave them feeling resentful and makes their tracking efforts feel futile. |
|  | Negative: not sharing with clinician due to fear of mental health services | Sometimes participants did not complete their life charts honestly because they feared there might be negative consequences such as a hospital admission. |
|  | Negative: sharing with clinician feels invasive | This [feature] would need to be optional; I’d just feel like Big Brother was watching me. |
|  | Positive: analysing data with family/friends | My partner and I use the graphs to look back at the prior week and attempt to identify any reasons for the changes in my mood. |
|  | Positive: sharing helps with planning and monitoring | There was a lot of good information in there...to be more reflective of what’s going on... and to involve people more directly, specifically my daughter. |
|  | Positive: sharing improves understanding and communication | They appreciated the association of pattern with behaviour. Made me feel more understood. |
|  | Positive: sharing with clinician is easier for me and them | During therapy or something, it [ESM] might also be very easy. Then the system could directly inform your clinician, rather than bringing a copy yourself, so to speak. That they [the clinicians] could directly, if you give your consent, have insight in the data. And yes, the system doesn’t lie. You can show that you have filled it out, at those moments. |
|  | Positive: facilitates discussions with clinician | If something is starting to get wonky... it’s easier when you have to talk to your psychiatrist because you can bring it in and say “this is what’s going on, this area is getting affected, these areas are getting affected”. |
|  | Positive: monitor and aid treatment | It is an easy way to convey a lot of information on one page. I show my GP on every visit, helps to look at how my medication is working against my mood swings. Aid discussion of relationships with other factors I have used these to discuss my migraines. Aid discussion of mood patterns We have both found them helpful particularly in identifying a moderately severe mixed mood state last year. |
| Clinician barriers and clinician facilitators | Barrier: worsens patient mood | On the Life Chart you can indicate that you score this or that, on average. A lot of people will then say that the actual situation is very different. So the micro-level is much more fine-grained. The danger is, though, that if people feel very bad, because their relationship has ended or I don’t know, that they will immediately think that they have a depression. That the micro-level overshadows the macro-level. |
|  | Barrier: burden of mood monitoring | Maybe. Maybe it was too much, but you don’t know that before- hand. That’s why I think: you have to try. And self-management is a major step. So to invest a good amount of energy into that, because you have a severe disorder, you can invest a lot of energy into that. |
|  | Barrier: concern about risk/liability issues | Well, I was really busy then, and then I also got those alerts and I thought: “do I have to do something with this as well?” That felt a bit as a responsibility, in a way. |
|  | Barrier: difficult to interpret results | If you look at the results, it really is so hard to interpret them. It’s still much more complicated than you had hoped beforehand. On the one hand, it’s a lot of data and I like graphs and such, I think they’re nice, you have a sort of overview, and well, about activities and such, it is solid. But what comes out as predictors disappoints me. Such that I think: it’s not so unequivocal or it’s not so easy to predict. Especially for people who are so instable in their mood, then the story gets even more unclear. |
|  | Barrier: self-appraisal of mood can be unreliable | There are people, if you ask them a number between 1 and 10 to indicate their stress level, who will say a 10 with a very calm demeanor. Or the other way around, sitting there like this [raising arms to indicate high stress level] saying, it’s a 5. An app like this [ESM] will have it wrong too, people are not so good at judging themselves. |
|  | Facilitator: helps guide treatment and medication response | With this they said they're able to get an overview of how the conditions are doing, the potential triggers and also if medication needs to be adjusted or if talking therapy refreshers would be useful. |
|  | Facilitator: prompts behaviour change | That is what I believe to be the advantage of self-monitoring and assignments you can do at home, outside our conversations here in the clinic: that you can adapt your own behavior and make healthy choices, so that is a nice side effect of this study, I think. |
|  | Facilitator: personalisation | I would really try to develop it tailored to the situation of the patient. And maybe link it to the relapse prevention plan. And it would be even better to also link it to the Life Chart method, for example. Or a sort of mood app, right? I mean, those exist, but are usually not so comprehensive. This way, you have all the information that you could use in treatment, and you have the aspect of self-management that can directly, in that moment, be adapted or stimulated even. |

Table S2: Sub-themes of adverse effects of mood tracking. x denotes endorsement of sub-theme in the study.

| **Source paper** | **Worsens mood and/or anxiety** | **Unhelpful reminder of mental illness** | **Decreases sense of autonomy** | **Limits ability to self-manage** | **Mood monitoring feels confronting** | **Mood monitoring can become compulsive** | **Mood monitoring feels like a burden** |
| --- | --- | --- | --- | --- | --- | --- | --- |
| Bos et al 2019 |  | x |  |  |  |  | x |
| Bos et al 2020 | x | x | x |  | x | x | x |
| Geerling et al 2021 | x | x |  |  |  | x |  |
| Jonathan et al 2021 2 |  |  |  |  |  |  |  |
| Jonathan et al 2021 |  |  |  |  |  |  |  |
| Jonathan et al 2024 |  |  |  |  |  |  | x |
| Gordon-Smith et al 2023 | x | x |  |  |  |  |  |
| Morton et al 2019 |  |  |  |  |  |  |  |
| Morton et al 2022 |  |  |  |  |  |  |  |
| Murnane et al 2015 | x |  |  |  |  |  | x |
| Murray et al 2011 |  |  |  |  |  |  | x |
| Nicholas et al 2017 |  |  |  |  | x |  |  |
| Rusch et al 2022 |  |  |  |  |  |  |  |
| Saunders et al 2017 | x |  | x |  |  |  |  |
| Sharma et al 2022 |  |  |  |  |  |  |  |
| Stern & Sin 2012 |  |  |  |  |  |  |  |
| Suto et al 2009 |  |  |  |  |  |  |  |
| Todd et al 2012 |  |  |  |  |  |  |  |
| van Bendegem et al 2014 | x | x | x | x | x |  | x |
| Van der Watt et al 2018 | x |  |  |  |  |  | x |

Table S3: Sub-themes of barriers to mood monitoring. x denotes endorsement of sub-theme in the study.

| **Source paper** | **Inconvenience of active ambulatory assessment** | **Adverse effects** | **No additional benefit** | **Data security concerns** | **Difficulties interpreting the data** | **Difficult to track mood when unwell** | **Requires a lot of commitment and energy** | **Mood monitoring feels impersonal** | **Lack interest in tracking when well** | **Mood monitoring superficial and restrictive** | **Repetitive nature of mood monitoring** | **Technical problems** | **Mood monitoring unreliable judge of mood** | **Use at the right stage of your illness** |
| --- | --- | --- | --- | --- | --- | --- | --- | --- | --- | --- | --- | --- | --- | --- |
| Bos et al 2019 |  |  | x |  |  |  |  |  |  |  |  |  |  | x |
| Bos et al 2020 | x | x |  |  | x |  |  |  |  |  |  |  | x |  |
| Geerling et al 2021 |  | x |  | x |  |  |  |  |  |  |  |  |  |  |
| Jonathan et al 2021 2 |  |  |  |  |  |  |  |  |  |  |  |  |  |  |
| Jonathan et al 2021 |  |  |  |  |  |  |  |  |  |  | x | x |  |  |
| Jonathan et al 2024 | x |  | x | x |  |  | x |  | x |  | x | x |  |  |
| Gordon-Smith et al 2023 |  | x | x |  | x | x |  |  |  | x |  | x |  |  |
| Morton et al 2019 |  |  |  |  |  |  |  |  |  | x |  |  |  |  |
| Morton et al 2022 |  |  |  |  |  |  |  |  |  |  |  |  |  |  |
| Murnane et al 2015 |  | x |  |  | x |  |  |  |  |  |  | x |  |  |
| Murray et al 2011 |  | x |  |  |  |  | x |  |  |  |  |  |  |  |
| Nicholas et al 2017 |  | x |  | x |  |  |  |  |  |  |  |  |  |  |
| Rusch et al 2022 |  |  |  |  |  |  |  |  |  |  | x |  |  |  |
| Saunders et al 2017 |  | x |  | x | x | x |  |  |  |  | x | x | x |  |
| Sharma et al 2022 |  |  |  |  |  |  |  |  |  |  |  |  |  |  |
| Stern & Sin 2012 |  |  |  |  |  |  |  |  |  |  |  |  |  |  |
| Suto et al 2009 |  |  |  |  |  |  | x |  |  |  |  |  |  |  |
| Todd et al 2012 |  |  |  |  |  |  |  |  |  |  |  |  |  |  |
| van Bendegem et al 2014 |  | x | x | x |  | x | x |  | x |  |  |  | x | x |
| Van der Watt et al 2018 |  | x | x |  |  | x |  | x |  |  | x |  |  |  |

Table S4: Sub-themes of facilitators to mood monitoring x denotes endorsement of sub-theme in the study.

| **Source paper** | **Advantages of passive ambulatory assessment** | **Already using a variety of mood monitoring methods** | **Active ambulatory assessment not over-burdening** | **Mood monitoring prompted behaviour change** | **Wearables well tolerated** |
| --- | --- | --- | --- | --- | --- |
| Bos et al 2019 | x |  |  |  |  |
| Bos et al 2020 |  |  | x |  |  |
| Geerling et al 2021 |  |  |  |  |  |
| Jonathan et al 2021 2 |  |  |  | x |  |
| Jonathan et al 2021 |  |  |  |  |  |
| Jonathan et al 2024 |  |  |  | x |  |
| Gordon-Smith et al 2023 |  |  |  |  |  |
| Morton et al 2019 |  |  |  |  |  |
| Morton et al 2022 |  |  |  |  |  |
| Murnane et al 2015 | x | x |  |  |  |
| Murray et al 2011 |  | x |  |  |  |
| Nicholas et al 2017 |  | x |  |  |  |
| Rusch et al 2022 | x |  |  |  |  |
| Saunders et al 2017 |  |  | x | x | x |
| Sharma et al 2022 |  |  |  |  |  |
| Stern & Sin 2012 |  |  |  |  |  |
| Suto et al 2009 |  |  |  | x |  |
| Todd et al 2012 |  | x |  |  |  |
| van Bendegem et al 2014 |  |  |  | x |  |
| Van der Watt et al 2018 |  |  |  |  |  |

Table S5: Sub-themes of desired features of the app. x denotes endorsement of sub-theme in the study.

| **Source paper** | **Other aspects in addition to mood monitoring** | **Crisis/wellness plan** | **Data transparency** | **Preference for digital methods** | **Effectiveness/scientific evaluation** | **Ease of use** | **Full control over many aspects of the process/app** | **Graphical display of mood** | **Medication reminder notifications** | **Reminder notifications to complete ambulatory assessment** | **Personalisation** | **Positive messages** | **Support required prior to/during use** | **Tracking additional features other than mood** |
| --- | --- | --- | --- | --- | --- | --- | --- | --- | --- | --- | --- | --- | --- | --- |
| Bos et al 2019 |  |  |  | x | x |  |  | x |  |  |  |  |  |  |
| Bos et al 2020 |  | x |  |  |  |  |  |  |  |  |  |  |  |  |
| Geerling et al 2021 | x | x | x |  |  | x | x | x |  | x | x | x |  |  |
| Jonathan et al 2021 2 |  | x |  |  |  |  |  |  | x |  | x |  | x | x |
| Jonathan et al 2021 | x | x |  |  | x | x |  |  |  |  | x |  | x | x |
| Jonathan et al 2024 | x |  |  |  | x | x |  |  |  | x | x |  | x | x |
| Gordon-Smith et al 2023 |  |  |  |  |  | x |  | x |  |  |  |  | x |  |
| Morton et al 2019 |  |  |  |  |  |  |  |  |  |  | x | x | x |  |
| Morton et al 2022 |  |  |  |  |  |  |  |  |  |  |  |  |  |  |
| Murnane et al 2015 |  |  |  | x |  |  | x |  |  |  | x |  |  | x |
| Murray et al 2011 |  |  |  |  |  |  |  |  |  |  | x |  | x |  |
| Nicholas et al 2017 |  | x | x |  | x | x | x |  |  | x | x | x |  |  |
| Rusch et al 2022 | x |  |  |  |  |  | x |  | x |  | x |  |  | x |
| Saunders et al 2017 | x |  | x |  |  | x | x | x |  |  | x |  |  | x |
| Sharma et al 2022 |  |  |  |  |  |  |  | x |  | x |  |  |  |  |
| Stern & Sin 2012 |  |  |  |  |  |  |  |  |  |  |  |  |  |  |
| Suto et al 2009 |  | x |  |  |  |  |  |  |  |  |  |  |  |  |
| Todd et al 2012 |  | x |  | x |  |  |  |  |  |  |  |  | x |  |
| van Bendegem et al 2014 |  |  | x |  |  |  | x | x |  |  | x |  | x |  |
| Van der Watt et al 2018 |  |  |  |  |  |  |  |  |  |  |  |  | x |  |

Table S6: Sub-themes of purpose of mood monitoring. x denotes endorsement of sub-theme in the study.

| **Source paper** | **Aid/monitor treatment** | **Improve communication** | **Early warning signs** | **Reassurance** | **Improves autonomy** | **Improve insight** | **Monitor long term mood** | **Monitor medication adherence** | **Monitor sleep** | **Improve motivation** | **Objectively judge mood** | **Relapse prevention** | **Helps stick to routine** | **Improves self-management** | **Periods when tracking more important** | **Use mood monitoring in a variety of different ways** |
| --- | --- | --- | --- | --- | --- | --- | --- | --- | --- | --- | --- | --- | --- | --- | --- | --- |
| Bos et al 2019 |  |  |  |  |  |  |  |  |  |  | x | x |  |  |  |  |
| Bos et al 2020 | x |  |  |  |  | x |  |  |  |  | x | x |  | x |  |  |
| Geerling et al 2021 |  |  |  |  |  | x |  |  |  |  |  | x |  |  |  |  |
| Jonathan et al 2021 2 |  |  | x | x |  | x |  | x | x | x | x | x | x | x |  |  |
| Jonathan et al 2021 |  |  |  |  |  | x |  |  | x | x | x |  |  | x |  |  |
| Jonathan et al 2024 |  |  |  |  |  |  |  |  | x |  |  |  | x |  |  |  |
| Gordon-Smith et al 2023 | x | x | x | x |  | x | x |  | x |  | x |  |  |  |  |  |
| Morton et al 2019 | x |  | x | x |  | x | x |  |  | x | x | x |  | x |  |  |
| Morton et al 2022 |  |  |  |  |  |  |  |  |  |  |  |  |  |  |  |  |
| Murnane et al 2015 | x | x |  |  |  | x |  |  |  |  |  |  |  | x | x |  |
| Murray et al 2011 | x |  | x |  | x | x |  |  | x |  |  |  | x | x |  |  |
| Nicholas et al 2017 |  |  | x |  |  |  |  |  | x |  |  |  |  | x |  |  |
| Rusch et al 2022 | x |  | x |  |  | x | x |  | x |  | x |  |  | x |  |  |
| Saunders et al 2017 | x |  |  |  | x | x | x |  | x |  | x |  |  | x |  |  |
| Sharma et al 2022 |  |  |  |  |  |  |  |  |  |  |  |  |  |  |  |  |
| Stern & Sin 2012 |  |  |  |  |  |  |  |  |  |  |  |  |  |  |  |  |
| Suto et al 2009 | x |  | x |  | x | x |  |  |  |  |  |  |  | x |  |  |
| Todd et al 2012 |  |  | x |  |  | x |  |  |  |  | x | x |  | x |  |  |
| van Bendegem et al 2014 | x | x | x |  |  | x | x |  |  |  | x | x |  | x |  | x |
| Van der Watt et al 2018 | x |  |  | x | x | x | x |  | x |  |  |  |  | x |  |  |

Table S7: Sub-themes of sharing with others (negative and positive). x denotes endorsement of sub-theme in the study.

| **Source paper** | **Negative: don’t want to burden others** | **Negative: others don’t understand** | **Negative: don’t want to medicalise other relationships** | **Negative: others overreact to the information I share with them** | **Negative: I want to maintain my privacy** | **Negative: clinician is not interested in the information** | **Negative: not sharing with clinician due to fear of mental health services** | **Negative: sharing with clinician feels invasive** | **Positive: analysing data with family/friends** | **Positive: sharing helps with planning and monitoring** | **Positive: sharing improves understanding and communication** | **Positive: sharing with clinician is easier for me and them** | **Positive: facilitates discussions with clinician** | **Positive: monitor and aid treatment** |
| --- | --- | --- | --- | --- | --- | --- | --- | --- | --- | --- | --- | --- | --- | --- |
| Bos et al 2019 |  |  |  |  |  |  |  |  |  |  |  |  |  |  |
| Bos et al 2020 |  |  |  |  |  |  |  |  |  |  |  | x |  |  |
| Geerling et al 2021 |  |  |  |  |  |  |  |  |  |  |  |  |  |  |
| Jonathan et al 2021 2 |  |  |  |  |  |  |  |  |  | x |  |  |  |  |
| Jonathan et al 2021 |  |  |  |  |  |  |  |  |  |  |  |  |  |  |
| Jonathan et al 2024 |  | x |  |  |  |  |  |  | x |  |  |  |  |  |
| Gordon-Smith et al 2023 |  | x |  | x |  | x |  |  | x |  | x |  |  | x |
| Morton et al 2019 |  |  |  |  |  |  |  |  |  |  |  |  | x |  |
| Morton et al 2022 |  |  |  |  |  |  |  |  |  |  |  |  |  |  |
| Murnane et al 2015 |  |  |  |  |  | x |  |  |  |  |  |  |  |  |
| Murray et al 2011 |  |  |  |  |  |  |  |  |  |  |  |  |  |  |
| Nicholas et al 2017 |  |  |  |  |  |  |  |  |  |  |  |  |  |  |
| Rusch et al 2022 |  |  |  |  |  |  |  | x |  |  |  |  |  |  |
| Saunders et al 2017 |  |  |  |  |  |  |  | x |  |  |  |  |  |  |
| Sharma et al 2022 |  |  |  |  |  |  |  |  |  |  |  |  |  |  |
| Stern & Sin 2012 |  |  |  |  |  |  |  |  |  |  |  |  |  |  |
| Suto et al 2009 |  |  |  |  |  |  |  |  |  |  |  |  |  |  |
| Todd et al 2012 |  |  |  |  |  |  |  |  |  |  |  |  |  |  |
| van Bendegem et al 2014 | x |  | x |  | x |  | x | x |  |  |  |  |  |  |
| Van der Watt et al 2018 |  | x |  |  |  |  |  |  |  |  |  |  |  |  |
|  | | | | | | | | | | | | | | |

Table S8: Clinician barriers/concerns and clinician facilitators/suggestions. x denotes endorsement of sub-theme in the study.

| **Source paper** | **Barrier: worsens patient mood** | **Barrier: burden of mood monitoring** | **Barrier: concern about risk/liability issues** | **Barrier: difficult to interpret results** | **Barrier: self-appraisal of mood can be unreliable** | **Facilitator: helps guide treatment and medication response** | **Facilitator: prompts behaviour change** | **Facilitator: personalisation** | **Facilitator: improves clinician-patient communication** | **Facilitator: improves insight and understanding** | **Facilitator: link to relapse prevention/crisis plan** |
| --- | --- | --- | --- | --- | --- | --- | --- | --- | --- | --- | --- |
| Bos et al 2019 |  |  |  |  |  |  |  |  |  |  |  |
| Bos et al 2020 | x | x | x | x | x |  | x | x |  | x | x |
| Geerling et al 2021 |  |  |  |  |  |  |  |  |  |  |  |
| Jonathan et al 2021 2 |  |  |  |  |  |  |  |  |  |  |  |
| Jonathan et al 2021 |  |  |  |  |  |  |  |  |  |  |  |
| Jonathan et al 2024 |  |  |  |  |  |  |  |  |  |  |  |
| Gordon-Smith et al 2023 |  |  |  |  |  | x |  |  | x |  |  |
| Morton et al 2019 |  |  |  |  |  |  |  |  |  |  |  |
| Morton et al 2022 |  |  |  |  |  |  |  |  |  |  |  |
| Murnane et al 2015 |  |  |  | x |  |  |  |  |  |  |  |
| Murray et al 2011 |  |  |  |  |  |  |  |  |  |  |  |
| Nicholas et al 2017 |  |  |  |  |  |  |  |  |  |  |  |
| Rusch et al 2022 |  |  |  |  |  |  |  |  |  |  |  |
| Saunders et al 2017 |  |  |  |  |  |  |  |  |  |  |  |
| Sharma et al 2022 |  |  |  |  |  |  |  |  |  |  |  |
| Stern & Sin 2012 |  |  |  |  |  |  |  |  |  |  |  |
| Suto et al 2009 |  |  |  |  |  |  |  |  |  |  |  |
| Todd et al 2012 |  |  |  |  |  |  |  |  |  |  |  |
| van Bendegem et al 2014 |  |  |  |  |  |  |  |  |  |  |  |
| Van der Watt et al 2018 |  |  |  |  |  |  |  |  |  |  |  |

REFERENCES

1. Bos FM, Snippe E, Bruggeman R, Wichers M, van der Krieke L. Insights of patients and clinicians on the promise of the experience sampling method for psychiatric care. Psychiatr Serv. Nov 1, 2019;70(11):983-991. [doi: 10.1176/appi.ps.201900050] [Medline: 31434558]
2. Bos FM, Snippe E, Bruggeman R, Doornbos B, Wichers M, van der Krieke L. Recommendations for the use of long-term experience sampling in bipolar disorder care: a qualitative study of patient and clinician experiences. Int J Bipolar Disord. Dec 1, 2020;8(1):38. [doi: 10.1186/s40345-020-00201-5] [Medline: 33258015]
3. Geerling B, Kelders SM, Kupka RW, Stevens AWMM, Bohlmeijer ET. How to make online mood-monitoring in bipolar patients a success? A qualitative exploration of requirements. Int J Bipolar Disord. Dec 1, 2021;9(1):39. [doi: 10.1186/s40345-021-00244-2] [Medline: 34851456]
4. Jonathan GK, Dopke CA, Michaels T, et al. A smartphone-based self-management intervention for bipolar disorder (LiveWell): user-centered development approach. JMIR Ment Health. Apr 12, 2021;8(4):e20424. [doi: 10.2196/20424] [Medline: 33843607]
5. Jonathan GK, Dopke CA, Michaels T, et al. A smartphone-based self-management intervention for individuals with bipolar disorder (LiveWell): qualitative study on user experiences of the behavior change process. JMIR Ment Health. Nov 22, 2021;8(11):e32306. [doi: 10.2196/32306] [Medline: 34813488]
6. Jonathan GK, Abitante G, McBride A, et al. LiveWell, a smartphone-based self-management intervention for bipolar disorder: Intervention participation and usability analysis. J Affect Disord. Apr 1, 2024;350:926-936. [doi: 10.1016/j.jad.2024.01.099] [Medline: 38246280]
7. Gordon-Smith K, Saunders KEA, Morton T, et al. User perspectives on long-term remote active electronic self-monitoring of mood symptoms in bipolar spectrum disorders. J Affect Disord. Mar 1, 2023;324:325-333. [doi: 10.1016/j.jad.2022.12.090] [Medline: 36584706]
8. Morton E, Hole R, Murray G, Buzwell S, Michalak E. Experiences of a web-based quality of life self-monitoring tool for individuals with bipolar disorder: a qualitative exploration. JMIR Ment Health. Dec 4, 2019;6(12):e16121. [doi: 10.2196/16121] [Medline: 31799936]
9. Morton E, Nicholas J, Yang L, et al. Evaluating the quality, safety, and functionality of commonly used smartphone apps for bipolar disorder mood and sleep self-management. Int J Bipolar Disord. Apr 4, 2022;10(1):10. [doi: 10.1186/s40345-022-00256-6] [Medline: 35368207]
10. Murnane EL, Cosley D, Chang P, et al. Self-monitoring practices, attitudes, and needs of individuals with bipolar disorder: implications for the design of technologies to manage mental health. J Am Med Inform Assoc. May 2016;23(3):477-484. [doi: 10.1093/jamia/ocv165] [Medline: 26911822]
11. Murray G, Suto M, Hole R, Hale S, Amari E, Michalak EE. Self-management strategies used by “high functioning” individuals with bipolar disorder: from research to clinical practice. Clin Psychol Psychother. 2011;18(2):95-109. [doi: 10.1002/cpp.710] [Medline: 20572206]
12. Nicholas J, Boydell K, Christensen H. Beyond symptom monitoring: Consumer needs for bipolar disorder self-management using smartphones. Eur Psychiatry. Jul 2017;44:210-216. [doi: 10.1016/j.eurpsy.2017.05.023] [Medline: 28692910]
13. Rusch A, Carley I, Badola P, et al. Digital mental health interventions for chronic serious mental illness: Findings from a qualitative study on usability and scale-up of the Life Goals app for bipolar disorder. Front Digit Health. 2022;4:1033618. [doi: 10.3389/fdgth.2022.1033618] [Medline: 36479190]
14. Saunders KEA, Bilderbeck AC, Panchal P, Atkinson LZ, Geddes JR, Goodwin GM. Experiences of remote mood and activity monitoring in bipolar disorder: A qualitative study. Eur Psychiatry. Mar 2017;41:115-121. [doi: 10.1016/j.eurpsy.2016.11.005] [Medline: 28135594]
15. Sharma AN, Barron-Millar E, Gaskell M, et al. Technology matters: Collaboratively augmenting longitudinal monitoring (C.A.L.M) in bipolar disorder - co-design, co-production and evaluation of the alpha prototype app. Child Adolesc Ment Health. Nov 2022;27(4):427-429. [doi: 10.1111/camh.12548] [Medline: 35261176]
16. Stern T, Sin J. Implementing a structured psychosocial interventions group programme for people with bipolar disorder. J Psychiatr Ment Health Nurs. Mar 2012;19(2):180-189. [doi: 10.1111/j.1365-2850.2011.01816.x] [Medline: 22070393]
17. Suto M, Murray G, Hale S, Amari E, Michalak EE. What works for people with bipolar disorder? Tips from the experts. J Affect Disord. Jul 2010;124(1-2):76-84. [doi: 10.1016/j.jad.2009.11.004] [Medline: 19969370]
18. Todd NJ, Jones SH, Lobban FA. What do service users with bipolar disorder want from a web-based self-management intervention? A qualitative focus group study. Clin Psychol Psychother. 2013;20(6):531-543. [doi: 10.1002/cpp.1804] [Medline: 22715161]
19. van Bendegem MA, van den Heuvel SCGH, Kramer LJ, Goossens PJJ. Attitudes of patients with bipolar disorder toward the Life Chart Methodology: a phenomenological study. J Am Psychiatr Nurses Assoc. 2014;20(6):376-385. [doi: 10.1177/1078390314558420] [Medline: 25367897]
20. Van der Watt ASJ, Roos T, Beyer C, Seedat S. Participants’ perspectives of weekly telephonic mood monitoring in South Africa: a feasibility study. Pilot Feasibility Stud. 2018;4:56. [doi: 10.1186/s40814-018-0245-0] [Medline: 29484200]

Information Section S1: Bipolar/depression mood tracking systematic review search strategy

Search performed 3/3/23. The search strategy was trialled on one database first and then refined subsequently. The search results were uploaded to Rayyan (71). Search terms were determined based on discussion between researchers, previous reviews and consultation with specialist librarians. The search was performed from inception to 3/3/23. The search was updated on 28/10/24.

**Number of abstracts original search 3/3/23:**

Medline: 2984

Embase: 4827

PsychINFO: 3346

SCOPUS: 2321

IEE Xplore: 615

Proquest dissertations and theses global: 2697

Proquest SciTech Collection: 3489

Total: 20,279

Full text review: 565

**Number of abstracts of updated search 3/3/24 – 28/10/24:**

Medline: 494

Embase: 920

PsychINFO: 364

SCOPUS: 1088

IEE Xplore: 99

Proquest dissertations and theses global: 0

Proquest SciTech Collection: 469

Total prior to deduplication: 3236

Auto-deduplicated: 1119

Total: 2117

Full text review: 193

**Published literature:**

| **OVID Medline** | |
| --- | --- |
| 1 | exp bipolar disorder/ OR exp depression OR exp mania/ |
| 2 | (((bipolar or bi polar) adj5 (disorder$ or depress$)) or ((cyclothymi$ or rapid or ultradian) adj5 cycl$) or hypomani$ or mania$ or manic$ or mixed episode$ or rcbd).mp |
| 3 | ('Depressive Disorder' OR 'Depression' OR 'dysthymi*' OR 'affective disorder' OR 'affective disorders' OR 'mood disorder' OR 'mood disorders' OR 'depression*' OR 'depressive*' OR 'dysthymic disorder').mp |
| 4 | 1 OR 2 OR 3 |
| 5 | ('self monitor*' or 'self assess*' or 'self manag*' or 'self record*' or 'self surveillance' or 'patient* monitor*' or 'measurement technolog*' or 'telemonitor*' or 'remote monitor*' or 'passive monitor*' or 'active monitor*' or 'mood track*' or 'mood monitor*' or 'experience sampl*' or 'ecological momentary assessment').mp |
| 6 | 4 adj10 5 |

<https://ovidsp.ovid.com/ovidweb.cgi?T=JS&NEWS=N&PAGE=main&SHAREDSEARCHID=10Q4IjupCc3HoHvVXInK959r2tcDy9vTlJlQsifUVfSVSyNrczwzGqVCqql3svtpo>

| **OVID EMBASE** | |
| --- | --- |
| 1 | bipolar disorder/ or bipolar depression/ or bipolar I disorder/ |
| 2 | depression assessment/ or treatment resistant depression/ or minor depression/ or chronic depression/ or postnatal depression/ or atypical depression/ or antenatal depression/ or adolescent depression/ or "mixed mania and depression"/ or post-stroke depression/ or endogenous depression/ or major depression/ or recurrent brief depression/ or depression/ or bipolar depression/ or perinatal depression/ or agitated depression/ or organic depression/ |
| 3 | "mixed mania and depression"/ or mania/ or bipolar mania/ |
| 4 | (((bipolar or bi polar) adj5 (disorder$ or depress$)) or ((cyclothymi$ or rapid or ultradian) adj5 cycl$) or hypomani$ or mania$ or manic$ or mixed episode$ or rcbd).mp. |
| 5 | ('Depressive Disorder' or 'Depression' or 'dysthymi*' or 'affective disorder' or 'affective disorders' or 'mood disorder' or 'mood disorders' or 'depression*' or 'depressive*' or 'dysthymic disorder').mp. |
| 6 | 1 OR 2 OR 3 OR 4 OR 5 |
| 7 | ('self monitor*' or 'self assess*' or 'self manag*' or 'self record*' or 'self surveillance' or 'patient* monitor*' or 'measurement technolog*' or 'telemonitor*' or 'remote monitor*' or 'passive monitor*' or 'active monitor*' or 'mood track*' or 'mood monitor*' or 'experience sampl*' or 'ecological momentary assessment').mp. |
| 8 | 6 adj10 7 |

https://ovidsp.ovid.com/ovidweb.cgi?T=JS&NEWS=N&PAGE=main&SHAREDSEARCHID=2Ofrc9VijRp6L40USOJFeEn3I1bHOmCW8O1Hzfz13xklneo3jW1767QyyDQMHnVDj

| **OVID PsychINFO** | |
| --- | --- |
| 1 | Bipolar Disorder/ or Bipolar II Disorder/ or Bipolar I Disorder/ or Mania/ |
| 2 | Major Depression/ or Endogenous Depression/ or Postpartum Depression/ or Recurrent Depression/ or "Depression (Emotion)"/ or Reactive Depression/ or Late Life Depression/ or Atypical Depression/ or Treatment Resistant Depression/ or "Long-term Depression (Neuronal)".mp. |
| 3 | (((bipolar or bi polar) adj5 (disorder$ or depress$)) or ((cyclothymi$ or rapid or ultradian) adj5 cycl$) or hypomani$ or mania$ or manic$ or mixed episode$ or rcbd).mp |
| 4 | 'Depressive Disorder' OR 'Depression' OR 'dysthymi*' OR 'affective disorder' OR 'affective disorders' OR 'mood disorder' OR 'mood disorders' OR 'depression*' OR 'depressive*' OR 'dysthymic disorder' |
| 5 | 1 OR 2 OR 3 OR 4 |
| 6 | ('self monitor*' or 'self assess*' or 'self manag*' or 'self record*' or 'self surveillance' or 'patient* monitor*' or 'measurement technolog*' or 'telemonitor*' or 'remote monitor*' or 'passive monitor*' or 'active monitor*' or 'mood track*' or 'mood monitor*' or 'experience sampl*' or 'ecological momentary assessment') |
| 7 | 5 adj10 6 |

https://ovidsp.ovid.com/ovidweb.cgi?T=JS&NEWS=N&PAGE=main&SHAREDSEARCHID=7WPhKe8RR9Athylx2jCCPdAkgbQlgcrdVpxl6NGPhskh73E8wr3X16vfACAP9Q54Y

**SCOPUS:**

TITLE-ABS-KEY({self monitor*} OR {self-monitor*} OR {self-assess*} OR {self manag*} OR {self-manag*} OR {self record*} OR {self-record*} OR {self surveillance} OR {self-surveillance} OR {patient* monitor*} OR {patient*-monitor*} OR {measurement technolog*} OR {measurement-technolog*} OR {telemonitor*} OR {remote monitor*} OR {remote-monitor*} OR {passive monitor*} OR {passive-monitor*} OR {active monitor*} OR {active-monitor*} OR {mood track*} OR {mood-track*} OR {mood monitor*} OR {mood-monitor*} OR {experience sampl*} OR {ecological momentary assessment}) W/10 ({Bipolar disorder*} OR {Manic depress*} OR {Manic-depress*} OR {Bipolar affective psychos*} OR {Bipolar depress*} OR {Manic disorder*} OR (72) OR {depressive disorder*} OR {major depressive disorder*} OR (72) OR {affective disorder*} OR {mood disorder*})

**IEE XPLORE:**

('self monitor' OR 'self monitoring' OR 'self assess' OR 'self assessment' OR 'self manage' OR 'self management' OR 'self record' OR 'self recording' OR 'self surveillance' OR 'patient monitor' OR 'patient monitoring' OR 'measurement technology' OR 'telemonitor' OR 'telemonitoring' OR 'remote monitor' OR 'remote monitoring' OR 'passive monitor' OR 'passive monitoring' OR 'active monitor*' OR 'mood track*' OR 'mood monitor*' OR 'experience sample' OR 'experience sampling' OR 'ecological momentary assessment') NEAR/10 ('Bipolar*' OR 'Manic disorder*' OR 'depressi*' OR 'affective disorder*' OR 'mood disorder*')

**Google scholar search:** An additional search of the first 15 pages of Google Scholar was conducted (search terms: ‘mood track’, ‘ecological momentary assessment’, ‘monitoring’, ‘remote monitoring’, ‘active monitor’, ‘passive monitor’, ‘experience sample’, ‘experience sampling’)

Finally, subject experts were approached to identify additional articles.

**Grey Literature:**

**ProQuest Dissertations & Theses Global:**

(("self monitor" OR "self monitoring" OR "self monitors") OR ("self assess" OR "self assessed" OR "self assessment") OR ("self manage" OR "self managed" OR "self managing") OR ("self record" OR "self recorded" OR "self recording") OR “self surveillance” OR “patient* monitor*” OR ("measurement technologies" OR "measurement technology") OR “telemonitor*” OR ("remote monitoring") OR ("passive monitoring") OR ("active monitoring") OR “mood track*” OR “mood monitor*” OR ("experience sampling") OR “ecological momentary assessment”) NEAR/10 (("bipolar disorder" OR "bipolar disorders") OR ("manic depression" OR "manic depressive") OR “Bipolar affective psychos*” OR ("bipolar depression") OR “Manic disorder*” OR “depressi*” OR ("depressive disorder") OR “major depressive disorder*” OR “depression” OR ("affective disorder" OR "affective disorders") OR ("mood disorder" OR "mood disorders"))

<http://abc.cardiff.ac.uk/login?url=https://www.proquest.com/search/2332884?accountid=9883>

<https://www.proquest.com/pqdtglobal>

**ProQuest SciTech Premium Collection:**

(("self monitor" OR "self monitoring" OR "self monitors") OR ("self assess" OR "self assessed" OR "self assessment") OR ("self manage" OR "self managed" OR "self managing") OR ("self record" OR "self recorded" OR "self recording") OR “self surveillance” OR “patient* monitor*” OR ("measurement technologies" OR "measurement technology") OR “telemonitor*” OR ("remote monitoring") OR ("passive monitoring") OR ("active monitoring") OR “mood track*” OR “mood monitor*” OR ("experience sampling") OR “ecological momentary assessment”) NEAR/10 (("bipolar disorder" OR "bipolar disorders") OR ("manic depression" OR "manic depressive") OR “Bipolar affective psychos*” OR ("bipolar depression") OR “Manic disorder*” OR “depressi*” OR ("depressive disorder") OR “major depressive disorder*” OR “depression” OR ("affective disorder" OR "affective disorders") OR ("mood disorder" OR "mood disorders"))

http://nottingham.idm.oclc.org/login?url=https://www.proquest.com/search/2332894?accountid=8018

<https://www.proquest.com/scitechpremium/>

**Google incognito mode – first 200 results:**

No new papers identified

**Systematic Reviews reference checked:**

These are cited in the main paper
