# Supplementary material for: The User Experience of Ambulatory Assessment and Mood Monitoring in Bipolar Disorder: Systematic Review and Meta-Synthesis of Qualitative Studies
Source: J Med Internet Res. 2025 Oct 17;27:e71525. doi: 10.2196/71525 (PMC12533931; doi:10.2196/71525)
Supplement: Multimedia Appendix 2 [file jmir-v27-e71525-s002.docx]

# Table S1: Characteristics of included studies.

| **Study** | **Country** | **Sample** | **n** | **Mean age in years for bipolar disorder (SD)** | **% female** | **Intervention** | **Setting** | **Active vs passive EMA** | **EMA or Mood Monitoring** | **EMA/Mood tracking  Procedure** | **EMA Duration** | **Generalisation vs personalisation** | **Person with BD led vs clinician led protocol** | **Time point of data collection** | **Data collection method** | **Data analysis method** |
| --- | --- | --- | --- | --- | --- | --- | --- | --- | --- | --- | --- | --- | --- | --- | --- | --- |
| Bos et al 2019 | Netherlands | Depression: 45%, Bipolar Disorder: 32%, Anxiety disorder: 18%, Psychosis: 14%, Eating disorder: 5%, Autism spectrum disorder: 5%, Unknown: 5% | Patients: 22, Clinicians: 21 | 20-35 years, n = 6, 36-50 years, n = 7, 51-65, n = 7, 66 or older, n = 2 | 64 | Hypothetical mood monitoring app | Mixed sample – participants recruited via secondary care outpatients and mental health institutions. | Active | Mood monitoring | Hypothetical app where individuals can record their moods, experiences, behaviors, contexts, and thoughts multiple times per day on their smart phones. | Hypothetical use | Personalisation | Clinician led | Hypothetical use | Semi-structured interview | Thematic analysis |
| Bos et al 2020 | Netherlands | Bipolar 1: 45%, Bipolar 2: 55% | Bipolar disorder: 18, Clinicians: 6 | 20-35 years: n = 9, 36-50 years: n = 8, 51-65 years: n = 3 | 80 | 5x EMA smartphone assessments daily - 29 items assessing monetary mood, symptoms, sleep and activities. Weekly ASRM, QIDS-SR-16 delivered via RoQua platform. | Tertiary care - mood disorders service | Active | EMA | 5x EMA smartphone assessments daily - 29 items assessing monetary mood, symptoms, sleep and activities. Weekly ASRM, QIDS-SR-16 delivered via RoQua platform. | 4 months (range: 16-32 weeks) | Personalisation | Person with BD led | End of study | Individual interviews | Thematic analysis |
| Geerling et al 2021 | Netherlands | Not reported | Bipolar disorder: 11, clinicians: 6 | 16-24 years: 1, 25-40: 4, 41-55: 5, 56-70: 3 | 53.8 | NIMH Life Chart Methodology - prospective — daily mood self-rating | Mixed sample – participants recruited via secondary care outpatients and advocacy groups | Active | Mood monitoring | NIMH Life Chart Methodology - prospective — daily mood self-rating | Variable depending on protocol | Generalisation | Clinician led | Not reported | Semi-structured focus groups | Deductive coding with discussion of concepts of a consensus document |
| Jonathan et al 2021 2 | USA | Not reported | 11 | 36 (14) | 63.6 | Livewell - smartphone based self management intervention | Secondary care – 1 previous mood episode in the past year and current care by psychiatrist/nurse practitioner | Active | Mood monitoring | Smartphone based self-management intervention - daily and weekly check-ins for weeks 1-16. Daily - adherence, sleep, duration, routine, wellness levels. Weekly - symptom severity scoring for all individual DSM-IV mood symptoms. | 8w | Personalisation | Person with BD led | End of study | Structured exit interview and exit questionnaire | Deductive coding guided by the interview script and the intervention’s behaviour change framework |
| Jonathan et al 2021 | USA | Not reported | 12 | 38 (14) | 66 | Livewell - smartphone based self management intervention | Secondary care | Active | Mood monitoring | 1. Simple smartphone app for self-monitoring of behavioural targets. 2.  LiveWell: Smartphone based self management intervention - daily and weekly check-ins for weeks 1-16. Daily - adherence, sleep, duration, routine, wellness levels. Weekly - symptom severity scoring for all individual DSM-IV mood symptoms. | Simple smartphone app: 12 weeks. LiveWell: 8 weeks. | Personalisation | Person with BD led | End of study | Structured exit interview and exit questionnaire | Instant data analysis, thematic analysis |
| Jonathan et al 2024 | USA | Bipolar 1: 100% | 17 | 45 (12) | 53 | Livewell - smartphone based self management intervention | Secondary care | Active | Mood monitoring | Smartphone based self-management intervention - daily and weekly check-ins for weeks 1-16. Daily - adherence, sleep, duration, routine, wellness levels. Weekly - symptom severity scoring for all individual DSM-IV mood symptoms. | 48 weeks | Personalisation | Person with BD led | End of study | Structured exit interview and exit questionnaire | Thematic analysis |
| Gordon-Smith et al 2023 | UK | Bipolar 1: 56.1%, Bipolar 2: 39.0%, Schizoaffective disorder - Bipolar type: 1.4%, Bipolar Disorder not otherwise specified: 3.6% | 362 | median: 54.0 (IQR: 17) | 67.4 | True Colours | Mixed sample – participants recruited via secondary care/advertising | Active | Mood monitoring | True Colours - ASRM/QIDS-SR16 delivered via weekly SMS/email | 28 months | Generalisation | Clinician led | After 28 months of use | Exit questionnaire | Content analysis |
| Morton et al 2019 | Canada | Bipolar 1: 55%, Bipolar 2: 37%, Bipolar 2 rapid cycling: 2.3%, Bipolar Disorder not otherwise specified: 4.7% | 43 | Modal age range: 45-54 | 69 | Web-based version of a BD-specific QoL self-report measure - the QoL tool | Mixed sample – participants recruited via tertiary care/advertising | Active | Mood monitoring | Web-based version of a BD-specific QoL self-report measure - weekly | Not reported | Generalisation | Person with BD led | End of study | Structured exit interview | Thematic analysis |
| Morton et al 2022 | Canada | Bipolar 1: 34.9%, Bipolar 2: 51.9%, Other/no formal diagnosis: 13.2% | 919 | 36.9 | 77.9 | Open enquiry into mood monitoring/tracking applications | Mixed sample – participants recruited via tertiary care/advertising | Active | Mood monitoring | Various mood/sleep monitoring/tracking applications: Daylio, Bipolar eMoods Tracker, iMood Journal, Moodpath, Calm, FitBit, Sleep Cycle, Samsung Health, Headspace. | Self-report previous use | Not clarified - various applications used | Not clarified - various applications used | End of study | Exit questionnaire | Descriptive statistics |
| Murnane et al 2015 | USA | Bipolar 1: 27%, Bipolar 2: 41%, Bipolar Disorder not otherwise specified: 17%, Cyclothymia: 2%, Not known: 9%, Declined: 4% (exact percentages not given) | 552 | Under 18: 1%, 18-24: 8%, 25-34: 24%, 35-44: 28%, 45-54: 23%, 55-64: 14%, 65-74: 2% (exact percentages not given) | 83 | Open enquiry into behaviour/health tracking practices | Mixed sample – participants recruited online | Active | Mood monitoring | Various mood/sleep monitoring/tracking applications: Charting software, digital notebooks, spreadsheets, smartphone applications, online mood trackers. | Self-report previous use | Not clarified - various applications used | Not clarified - various applications used | End of study | Survey including multiple-choice, multiple-answer, Likert-style and open-ended questions | Quantitative data analysed descriptively, responses to open-ended questions were qualitatively analysed using open coding |
| Murray et al 2011 | Canada | Bipolar 1: 78.1%, Bipolar 2: 21.9% | 33 | 41.1 (13.3) | 63 | Open enquiry into behaviour/health tracking practices | Mixed sample – participants recruited via advertising in person and online | Active | Mood monitoring | Various analogue mood/sleep monitoring/tracking strategies | Self-report previous use | Personalisation | Person with BD led | End of study | Individual or group interview | Thematic analysis |
| Nicholas et al 2017 | Australia | Not reported | 89 | 24.4 (3.9) | 87 | Open enquiry into mood monitoring/tracking applications | Mixed sample – participants recruited online | 16 different apps used for mood monitoring | Mood monitoring | Various mood/sleep monitoring/tracking applications | Self-report previous use | Not clarified - various applications used | Not clarified - various applications used | End of study | Cross-sectional survey regarding technology use, app use for self-management and app features important during app selection | Descriptive statistics and thematic analysis |
| Rusch et al 2022 | USA | Bipolar 1: 83.3%, Bipolar 2: 11.1% | total: 91, qualitative interviews: 18 | 49.2 (13.7) | 66.7 | Life Goals app | Existing cohort | Active | Mood monitoring | Life Goals app: mood monitoring symptoms of anxiety, depression and mania using the PHQ-9, GAD-7, Internal State Scale | 6 months | Personalisation | Clinician led | End of study | Semi-structured exit interview | Affinity mapping/Thematic analysis |
| Saunders et al 2017 | UK | Bipolar 1: 66.6%, Bipolar 2: 33.3% | 21 | 44.38 | 66.7 | True Colours | Mixed sample – participants recruited via secondary care/advertising | Active & passive EMA | Mood monitoring | True Colours - ASRM/QIDS-SR16 delivered via weekly SMS/email. Daily mood monitoring via smartphone app and wearables. One week of 10x daily EMA and continuous use of wearables. | 3 months | Generalisation | Clinician led | End of study | Semi-structured interview | Framework technique: thematic and case-based analysis |
| Sharma et al 2022 | UK | Not reported | 13 | Range: 14.5 to 24.2 | 92.3 | Collaboratively augmenting longitudinal monitoring (C.A.L.M) in Bipolar Disorder | Not reported | Active | Mood monitoring | CALM in Bipolar Disorder: mood monitoring | 3 months | Personalisation | Person with BD led | End of study | Semi-structured interview | Not reported |
| Stern & Sin 2012 | UK | Not reported | 23 | Not reported | Not reported | Structured Group Psychosocial Intervention | Secondary care | Active | Mood monitoring | Structured Group Psychosocial Intervention - incorporating mood monitoring | 3 months | Generalisation | Clinician led | End of study | Exit questionnaire | Descriptive statistics |
| Suto et al 2009 | Canada | Bipolar 1: 78.1%, Bipolar 2: 21.9% | 32 | 41.1 (13.3) | 63 | Open enquiry into behaviour/health tracking practices | Mixed sample – participants recruited via advertising in person and online | Active | Mood monitoring | Various self management strategies including monitoring | Self-report previous use | Personalisation | Person with BD led | End of study | Individual or group interview | Thematic analysis |
| Todd et al 2012 | UK | Bipolar 1: 66.6%, Bipolar 2: 33.3% | 12 | 42 | 41.6 | Living With Bipolar | Mixed sample – participants recruited via advertising in person and online | Active | Mood monitoring | Living With Bipolar - web-based self-management intervention | Exploratory study without testing of the intervention | Personalisation | Person with BD led | End of study | Focus groups | Thematic analysis |
| van Bendegem et al 2014 | Netherlands | Bipolar 1: 92.9%, Bipolar 2: 7.1% | 14 | 41.5 | 50 | NIMH Life Chart Methodology - prospective — daily mood self-rating | Secondary care | Active | Mood monitoring | NIMH Life Chart Methodology - prospective — daily mood self-rating | 2 months - 3 years | Generalisation | Clinician led | End of study | Semi-structured interview | Interpretative phenomenological analysis |
| Van der Watt et al 2018 | Netherlands | Bipolar Disorder: 35.1%, Depression: 56.8%, Mood and anxiety disorder: 2.7%, Other affective disorder: 5.4% | 37 | 35.76 (10.8) | 89.2 | Weekly telephone mood monitoring | Secondary care | Active | Mood monitoring | Weekly telephone mood monitoring | 6.5 months | Generalisation | Clinician led | End of study | Semi-structured interview | Thematic content analysis |

# Table S2: Risk of bias assessments for included studies (1 = present, 0 = absent) .

| **Study** | **Assessment of Study Quality** | | | | | | | | | |
| --- | --- | --- | --- | --- | --- | --- | --- | --- | --- | --- |
|  | Was there a clear statement of the aims of the research? | Is a qualitative methodology appropriate? | Was the research design appropriate to address the aims of the research? | Was the recruitment strategy appropriate to the aims of the research? | Was the data collected in a way that addressed the research issue? | Has the relationship between researcher and participants been adequately considered? | Have ethical issues been taken into consideration? | Was the data analysis sufficiently rigorous? | Is there a clear statement of findings? | How valuable is the research? |
| Bos et al 2019 | 1 | 1 | 1 | 1 | 1 | 0 | 1 | 1 | 1 | 1 |
| Bos et al 2020 | 1 | 1 | 1 | 1 | 1 | 0 | 1 | 1 | 1 | 1 |
| Geerling et al 2021 | 1 | 1 | 1 | 1 | 1 | 0 | 1 | 1 | 1 | 1 |
| Jonathan et al 2021 2 | 1 | 1 | 1 | 1 | 1 | 0 | 1 | 1 | 1 | 1 |
| Jonathan et al 2021 | 1 | 1 | 1 | 1 | 1 | 0 | 1 | 1 | 1 | 1 |
| Jonathan et al 2024 | 1 | 1 | 1 | 1 | 1 | 0 | 1 | 1 | 1 | 1 |
| Gordon-Smith et al 2023 | 1 | 1 | 1 | 1 | 1 | 0 | 1 | 1 | 1 | 1 |
| Morton et al 2019 | 1 | 1 | 1 | 1 | 1 | 0 | 1 | 1 | 1 | 1 |
| Morton et al 2022 | 1 | 1 | 1 | 1 | 1 | 0 | 1 | 1 | 1 | 1 |
| Murnane et al 2015 | 1 | 1 | 1 | 1 | 1 | 0 | 1 | 1 | 1 | 1 |
| Murray et al 2011 | 1 | 1 | 1 | 1 | 1 | 0 | 1 | 1 | 1 | 1 |
| Nicholas et al 2017 | 1 | 1 | 1 | 1 | 1 | 0 | 1 | 1 | 1 | 1 |
| Rusch et al 2022 | 1 | 1 | 1 | 1 | 1 | 0 | 1 | 1 | 1 | 1 |
| Saunders et al 2017 | **1** | 1 | 1 | 1 | 1 | 0 | 0 | 1 | 1 | 1 |
| Sharma et al 2022 | 1 | 1 | 1 | 1 | 1 | 0 | 1 | 0 | 1 | 1 |
| Stern & Sin 2012 | 1 | 1 | 1 | 1 | 1 | 0 | 0 | 1 | 1 | 1 |
| Suto et al 2009 | 1 | 1 | 1 | 1 | 1 | 0 | 1 | 1 | 1 | 1 |
| Todd et al 2012 | 1 | 1 | 1 | 1 | 1 | 0 | 1 | 1 | 1 | 1 |
| van Bendegem et al 2014 | 1 | 1 | 1 | 1 | 1 | 0 | 1 | 1 | 1 | 1 |
| Van der Watt et al 2018 | 1 | 1 | 1 | 1 | 1 | 0 | 1 | 1 | 1 | 1 |

REFERENCES

1. Bos FM, Snippe E, Bruggeman R, Wichers M, van der Krieke L. Insights of patients and clinicians on the promise of the experience sampling method for psychiatric care. Psychiatr Serv. Nov 1, 2019;70(11):983-991. [doi: 10.1176/appi.ps.201900050] [Medline: 31434558]
2. Bos FM, Snippe E, Bruggeman R, Doornbos B, Wichers M, van der Krieke L. Recommendations for the use of long-term experience sampling in bipolar disorder care: a qualitative study of patient and clinician experiences. Int J Bipolar Disord. Dec 1, 2020;8(1):38. [doi: 10.1186/s40345-020-00201-5] [Medline: 33258015]
3. Geerling B, Kelders SM, Kupka RW, Stevens AWMM, Bohlmeijer ET. How to make online mood-monitoring in bipolar patients a success? A qualitative exploration of requirements. Int J Bipolar Disord. Dec 1, 2021;9(1):39. [doi: 10.1186/s40345-021-00244-2] [Medline: 34851456]
4. Jonathan GK, Dopke CA, Michaels T, et al. A smartphone-based self-management intervention for bipolar disorder (LiveWell): user-centered development approach. JMIR Ment Health. Apr 12, 2021;8(4):e20424. [doi: 10.2196/20424] [Medline: 33843607]
5. Jonathan GK, Dopke CA, Michaels T, et al. A smartphone-based self-management intervention for individuals with bipolar disorder (LiveWell): qualitative study on user experiences of the behavior change process. JMIR Ment Health. Nov 22, 2021;8(11):e32306. [doi: 10.2196/32306] [Medline: 34813488]
6. Jonathan GK, Abitante G, McBride A, et al. LiveWell, a smartphone-based self-management intervention for bipolar disorder: Intervention participation and usability analysis. J Affect Disord. Apr 1, 2024;350:926-936. [doi: 10.1016/j.jad.2024.01.099] [Medline: 38246280]
7. Gordon-Smith K, Saunders KEA, Morton T, et al. User perspectives on long-term remote active electronic self-monitoring of mood symptoms in bipolar spectrum disorders. J Affect Disord. Mar 1, 2023;324:325-333. [doi: 10.1016/j.jad.2022.12.090] [Medline: 36584706]
8. Morton E, Hole R, Murray G, Buzwell S, Michalak E. Experiences of a web-based quality of life self-monitoring tool for individuals with bipolar disorder: a qualitative exploration. JMIR Ment Health. Dec 4, 2019;6(12):e16121. [doi: 10.2196/16121] [Medline: 31799936]
9. Morton E, Nicholas J, Yang L, et al. Evaluating the quality, safety, and functionality of commonly used smartphone apps for bipolar disorder mood and sleep self-management. Int J Bipolar Disord. Apr 4, 2022;10(1):10. [doi: 10.1186/s40345-022-00256-6] [Medline: 35368207]
10. Murnane EL, Cosley D, Chang P, et al. Self-monitoring practices, attitudes, and needs of individuals with bipolar disorder: implications for the design of technologies to manage mental health. J Am Med Inform Assoc. May 2016;23(3):477-484. [doi: 10.1093/jamia/ocv165] [Medline: 26911822]
11. Murray G, Suto M, Hole R, Hale S, Amari E, Michalak EE. Self-management strategies used by “high functioning” individuals with bipolar disorder: from research to clinical practice. Clin Psychol Psychother. 2011;18(2):95-109. [doi: 10.1002/cpp.710] [Medline: 20572206]
12. Nicholas J, Boydell K, Christensen H. Beyond symptom monitoring: Consumer needs for bipolar disorder self-management using smartphones. Eur Psychiatry. Jul 2017;44:210-216. [doi: 10.1016/j.eurpsy.2017.05.023] [Medline: 28692910]
13. Rusch A, Carley I, Badola P, et al. Digital mental health interventions for chronic serious mental illness: Findings from a qualitative study on usability and scale-up of the Life Goals app for bipolar disorder. Front Digit Health. 2022;4:1033618. [doi: 10.3389/fdgth.2022.1033618] [Medline: 36479190]
14. Saunders KEA, Bilderbeck AC, Panchal P, Atkinson LZ, Geddes JR, Goodwin GM. Experiences of remote mood and activity monitoring in bipolar disorder: A qualitative study. Eur Psychiatry. Mar 2017;41:115-121. [doi: 10.1016/j.eurpsy.2016.11.005] [Medline: 28135594]
15. Sharma AN, Barron-Millar E, Gaskell M, et al. Technology matters: Collaboratively augmenting longitudinal monitoring (C.A.L.M) in bipolar disorder - co-design, co-production and evaluation of the alpha prototype app. Child Adolesc Ment Health. Nov 2022;27(4):427-429. [doi: 10.1111/camh.12548] [Medline: 35261176]
16. Stern T, Sin J. Implementing a structured psychosocial interventions group programme for people with bipolar disorder. J Psychiatr Ment Health Nurs. Mar 2012;19(2):180-189. [doi: 10.1111/j.1365-2850.2011.01816.x] [Medline: 22070393]
17. Suto M, Murray G, Hale S, Amari E, Michalak EE. What works for people with bipolar disorder? Tips from the experts. J Affect Disord. Jul 2010;124(1-2):76-84. [doi: 10.1016/j.jad.2009.11.004] [Medline: 19969370]
18. Todd NJ, Jones SH, Lobban FA. What do service users with bipolar disorder want from a web-based self-management intervention? A qualitative focus group study. Clin Psychol Psychother. 2013;20(6):531-543. [doi: 10.1002/cpp.1804] [Medline: 22715161]
19. van Bendegem MA, van den Heuvel SCGH, Kramer LJ, Goossens PJJ. Attitudes of patients with bipolar disorder toward the Life Chart Methodology: a phenomenological study. J Am Psychiatr Nurses Assoc. 2014;20(6):376-385. [doi: 10.1177/1078390314558420] [Medline: 25367897]
20. Van der Watt ASJ, Roos T, Beyer C, Seedat S. Participants’ perspectives of weekly telephonic mood monitoring in South Africa: a feasibility study. Pilot Feasibility Stud. 2018;4:56. [doi: 10.1186/s40814-018-0245-0] [Medline: 29484200]
